# The Rab-binding Profiles of Bacterial Virulence Factors during Infection* [Fn FN2]

**DOI:** 10.1074/jbc.M115.700930

**Published:** 2016-01-11

**Authors:** Ernest C. So, Gunnar N. Schroeder, Danielle Carson, Corinna Mattheis, Aurélie Mousnier, Malgorzata Broncel, Edward W. Tate, Gad Frankel

**Affiliations:** From the ‡ MRC Centre for Molecular Bacteriology and Infection, Department of Life Sciences, Imperial College, London SW7 2AZ, United Kingdom,; § Department of Chemistry, South Kensington Campus, Imperial College, London SW7 2AZ, United Kingdom,; ¶ Department of Chemistry, Institute of Chemical Biology, Imperial College, London SW7 2AZ, United Kingdom

**Keywords:** bacterial pathogenesis, mass spectrometry (MS), protein complex, protein cross-linking, protein purification, *Legionella pneumophila*, LidA, Rab GTPases, SidM, Type IV secretion system effectors

## Abstract

*Legionella pneumophila*, the causative agent of Legionnaire's disease, uses its type IV secretion system to translocate over 300 effector proteins into host cells. These effectors subvert host cell signaling pathways to ensure bacterial proliferation. Despite their importance for pathogenesis, the roles of most of the effectors are yet to be characterized. Key to understanding the function of effectors is the identification of host proteins they bind during infection. We previously developed a novel tandem-affinity purification (TAP) approach using hexahistidine and BirA-specific biotinylation tags for isolating translocated effector complexes from infected cells whose composition were subsequently deciphered by mass spectrometry. Here we further advanced the workflow for the TAP approach and determined the infection-dependent interactomes of the effectors SidM and LidA, which were previously reported to promiscuously bind multiple Rab GTPases *in vitro*. In this study we defined a stringent subset of Rab GTPases targeted by SidM and LidA during infection, comprising of Rab1A, 1B, 6, and 10; in addition, LidA targets Rab14 and 18. Taken together, this study illustrates the power of this approach to profile the intracellular interactomes of bacterial effectors during infection.

## Introduction

Many bacterial pathogens use secretion systems to translocate virulence factors, termed effectors, into the host cell where they subvert cell signaling to facilitate bacterial survival and proliferation (1). Understanding the function of the effectors is central for understanding pathogenesis. However, prediction of effector functions using bioinformatics is of limited effectiveness as many effectors share little to no homology with known proteins. Therefore, key to deciphering effector function is the identification of the proteins they target during infection.

*Legionella pneumophila*, a Gram-negative pathogen, is the causative agent of Legionnaire's disease, a severe and potentially fatal pneumonia. Following entry into alveolar macrophages and epithelial cells, *Legionella* avoids lysosomal degradation and forms a replicative niche, the *Legionella* containing vacuole (LCV)^4^ (reviewed in So *et al.* (2)). The defect in organelle trafficking/intracellular multiplication (Dot/Icm) type IV secretion system (T4SS), which translocates over 300 mostly uncharacterized effectors, is essential for *Legionella*'s ability to survive and replicate intracellularly (3–6).

Central to the *Legionella* virulence strategy is its ability to manipulate the function of multiple Rab GTPases, which are important regulators of vesicular trafficking. Proteomic analysis of the LCV has shown that a large number of Rab GTPases (Rab1, 2, 4, 5, 6, 7, 8, 10, 11, 14, 18, 21, 31, and 32) are recruited to the LCV (7). In particular, manipulation of Rab1, involved in trafficking between the endoplasmic reticulum (ER) and the Golgi, has been elucidated in great detail. Critical to the manipulation of Rab1 is the effector SidM (DrrA), which contains three functional domains. Upon translocation, it anchors via a phosphatidylinositol 4-phosphate (PI4P) binding domain onto the LCV (8), where it recruits and activates Rab1 using its guanine nucleotide exchange factor (GEF) domain (9). SidM's adenylyltransferase domain modifies GTP-bound Rab1 with an AMP moiety (10), which locks Rab1 into a constitutively active state and prevents deactivation by the effector GTPase activating protein (GAP) LepB (11) until removal of the AMP group by the effector SidD (12, 13). In addition, the effector LidA was implicated in Rab1 recruitment to the LCV (14); however, its precise function is currently unknown.

Besides the well characterized interactions with Rab1, *in vitro* binding assays have shown that SidM can bind Rab8B, 10, 27A/B, 31, 35 while LidA can bind Rab2, 3B, 4B, 5, 6, 7, 8A/B, 9, 10, 11, 13, 14, 18, 20, 22, 27A/B, 30, 31, 32, and 35 (15, 16). In particular, LidA binds Rab1, 6, and 8 with picomolar affinities *in vitro* (17). This shows promiscuous Rab GTPase binding capacity of the two effectors; however, the selective distribution of Rab GTPases to specific subcellular compartments as well as the predominant localization of SidM and LidA on the LCV suggest that their interactions during infection might be governed by spatial mobility constraints. It is currently unknown which of these reported effector-Rab GTPase interactions are relevant during infection.

We have recently developed a novel method to isolate intracellular effector protein complexes during infection of cultured cells (18), enabling us to overcome the common pitfalls of *in vitro* assays which often deliver false-positives or miss interactors as they do not account for the unique microenvironment found in infected cells. In this assay, *Legionella* expressing an effector of interest fused to a hexahistidine (His_6_)-tag and a 15 amino acid residue BirA biotinylation site (Bio) infect host cells stably expressing BirA, the *Escherichia coli* biotin ligase. Provision of BirA only in the host cell allows enhanced discrimination of translocated effector from the remaining intrabacterial pool, enabling the reduction of bacterial background binders. In addition, as both affinity tags are small and inert to detergents and denaturing chemicals, the system allows the use of a wide range of effective lysis and purification conditions. Formaldehyde crosslinking prior to cell lysis allows protein-protein interactions to be stabilized. Tandem affinity purification (TAP) of effector complexes formed in infected cells using Ni^2+^ nitrilotriacetic acid (NTA) and streptavidin affinity purification permits the composition of effector complexes to be determined using liquid chromatography tandem mass spectrometry (LC-MS/MS). We first successfully employed the technique to study the transmembrane effector PieE and showed that it forms a multi-domain Rab GTPase binding hub on the LCV during infection. The aim of this project was to use optimized effector complex purification protocols and semi-quantitative proteomics to determine which Rab GTPases are targeted by SidM and LidA in infected cells.

## Experimental Procedures

### 

#### 

##### Molecular Biology

Bacterial strains used in this study are shown in Supplemental Table S1. Plasmids were constructed using standard molecular biology techniques with primers and restriction enzymes indicated in Supplemental Table S2. Chromosomal DNA from *L. pneumophila* strain 130b (ATCC BAA-74) served as template. GFP-Rab2a, -Rab5c, and -Rab10 were amplified from pENTR-Rab GTPase constructs as templates. GFP-BirA was amplified from pICC1394 as template (18). Sequence identities of the constructs were confirmed by DNA sequencing.

##### Tissue Culture

HEK293E, A549, A549-BirA (18), A549-GFP-Rab2a, -Rab5c, and -Rab10 cells were grown in Dulbecco's Modified Eagle Medium (DMEM) (Sigma) supplemented with 10% fetal calf serum (FCS) (Gibco), 1% GlutaMAX (Gibco), and non-essential amino acids (Sigma). THP-1 and THP-1-BirA cells were grown in RPMI (Sigma) supplemented with 10% FCS and 1% GlutaMAX. All cells were cultured under a humidified atmosphere (5% CO_2_, 37 °C).

##### Production of A549-GFP-Rab and THP-1-BirA Cell Lines

A549 and THP-1 cells were virally transduced as described previously (19). Briefly, a solution of pMXs-IP containing a GFP-Rab or GFP-BirA, pCMV-VSV-G envelope and pCMV-MMLV-gag-pol packaging plasmids was transfected into HEK293E cells using Lipofectamine 2000 (Life Technologies) according to the manufacturer's protocol. After 24 h, the medium was replaced and left for a further 24 h. The supernatant was collected, sterile filtered (0.45 μm) and added to cultured A549 or THP-1 cells. The A549 and THP-1 cells were selected with puromycin (Gibco) (1.5 μg/ml and 2 μg/ml, respectively) for at least 24 h and viable cells maintained in culture as described above. Transduced cells were sorted by flow cytometry to obtain a fluorescently homogenous population.

##### Legionella Culture

*L. pneumophila* strain 130b (ATCC BAA-74) was grown as described previously (20). For infection, bacteria grown for 3 days on buffered charcoal yeast extract (CYE) agar plates were resuspended to a starting OD of 0.1 in *N*-(2-acetamido)-2-aminoethanesulfonic acid (ACES)-buffered yeast extract (AYE) broth and incubated at 37 °C for 21 h. Chloramphenicol (Cm) was used at 6 μg/ml as required. Expression of tagged effectors was induced at 20 h post inoculation by addition of 1 mm isopropyl β-d-1-thiogalactopyranoside (IPTG) for 1 h.

##### Legionella Infection

A549-BirA cells were seeded at 4 × 10^6^ cells per 10-cm Petri dish for 14 h. THP-1-BirA cells were seeded at 1 × 10^7^ cells per 10 cm Petri dish and differentiation induced by addition of 80 nm phorbol 12-myristate 13-acetate (PMA) for 3 days. Prior to infection, 4 μm biotin (Sigma), 6 μg/ml Cm and 1 mm IPTG were added to the medium. A549-BirA and THP-1-BirA were infected with *L. pneumophila* strains at a multiplicity of infection (MOI) of 15 and 1 respectively. Cells were washed 2 h post-infection with 3 × 5 ml PBS and fresh medium (supplemented with 1% GlutaMAX, 10% FCS, 4 μm biotin, 6 μg/ml Cm, 1 mm IPTG) added and incubated for another 22 h (A549-BirA) or 4 h (THP-1-BirA).

##### Effector Complex Isolation and Processing for LC-MS/MS

Isolation of effector complexes was adapted from Mousnier *et al.* (18). All effector complex isolation experiments were performed in technical triplicate (SAP/TAP, THP-1, lysis buffers, formaldehyde concentrations, and LidA experiments) or biological duplicate of technical duplicates (crosslinker reactivity experiment). Composition of all buffers are shown in Supplemental Tables S3 and S4. All steps were performed at the lysis buffer dependent temperature indicated in Supplemental Table S4 unless otherwise stated. 6/24 h infected cells were washed with 2 × 5 ml PBS, fixed using 5-ml crosslinking solution for 30 min at room temperature and quenched by addition of 500 μl of 1.25 m glycine/50 mm l-cysteine in PBS for 15 min at room temperature. Cells were washed with 3 × 5 ml PBS and lysed in 1 ml of lysis buffer with protease inhibitors (Roche) and Benzonase (Novagen) for 30 min. Cells were scraped and insoluble fraction removed by centrifugation for 20 min at 20,000 × *g*.

The soluble fraction was added to pre-equilibrated Ni^2+^-NTA (Qiagen) (60 μl of 50% slurry per sample) and incubated for 1 h on a rotating wheel. The resin was washed 5 × 1 ml His wash buffer with 1000 × *g* 1 min spin at 4 °C in between. Bound complexes were eluted using 3 × 200 μl elution buffer (10 min on vortex shaker and 1000 × *g* 1 min spin at 4 °C to pellet the resin).

Elution fractions were combined, centrifuged at 20000 × *g* for 1 min at 4 °C, transferred to pre-equilibrated Neutravidin agarose (Pierce) (50 μl of 50% slurry per sample) and incubated at 4 °C for 2 h on a rotating wheel. The resin was washed with 4 × 1 ml Triton lysis buffer and 4 × 1 ml 50 mm ammonium bicarbonate (AMBIC). 50 μl of AMBIC was left over the resin after the final wash. Sequencing grade modified trypsin (1 μg) (Promega) was added to each sample and incubated at 37 °C overnight. Supernatants containing tryptic peptides were collected by addition of 1 × 80 μl AMBIC and 1 × 80 μl of 0.1% formic acid with 10 min vortex shaking and 3000 × *g* 2 min centrifugation.

Peptide mixtures were desalted by Stage-Tip method as described previously (21) and dimethyl labeled according to Boersema *et al.* (22). Bio and K/A samples were light and heavy dimethyl labeled, respectively. Briefly, peptide mixtures were loaded onto sorbent (SDB-XC poly(styrenedivinylbenzene) copolymer, from 3 m) and desalted with 150 μl of water. 100 μl of dimethyl labeling solution (Supplemental Table S5) were passed through the Stage-Tip over 30 min. The membrane was washed with 150 μl of water and peptides eluted using 79% acetonitrile. Samples were vacuum dried and stored at −80 °C. Samples were resuspended in 20 μl of 0.5% trifluoroacetic acid, 2% acetonitrile, and transferred into LC-MS sample vials.

##### Mass Spectrometry

The analysis was performed using an Acclaim PepMap RSLC column of 50 cm × 75 μm inner diameter (Thermo Fisher Scientific) using a 2 h acetonitrile gradient in 0.1% aqueous formic acid at a flow rate of 250 nl/min. Easy nLC-1000 was coupled to a Q Exactive mass spectrometer via an easy-spray source (all Thermo Fisher Scientific). The Q Exactive was operated in data-dependent mode with survey scans acquired at a resolution of 75,000 at *m*/*z* 200 (transient time 256 ms). Up to ten of the most abundant isotope patterns with charge +2 or higher from the survey scan were selected with an isolation window of 3.0 *m*/*z* and fragmented by higher-energy collisional dissociation with normalized collision energies of 25. The maximum ion injection times for the survey scan and the MS/MS scans (acquired with a resolution of 17,500 at *m*/*z* 200) were 20 and 120 ms, respectively. The ion target value for MS was set to 10^6^ and for MS/MS to 10^5^, and the intensity threshold was set to 8.3 × 10^2^.

##### MS Data Processing

The data were processed using MaxQuant (version 1.5.0.25) (23) and peptides were identified by matching MS/MS spectra with reference human (Uniprot, downloaded on 19/01/2015) and *L. pneumophila* strain 130b (ORF extraction of draft genome, Schroeder *et al.*, ftp://ftp.sanger.ac.uk/pub/pathogens/Legionella/pneumophila/130b/ (20)) proteomes using Andromeda search engine (24). N-terminal acetylation and methionine oxidation were selected as variable modifications. No fixed modifications were set. Reference proteomes were *in silico* digested using the trypsin/P setting whereby cleavages were allowed after arginine/lysine residues but only if it is not followed by a proline. Light (+28 Da) and heavy (+32 Da) dimethyl labeled lysines and N termini were used for quantification by a built-in algorithm in MaxQuant (23). Up to two missed cleavages were allowed. The false discovery rate was set to 0.01 for peptides, proteins, and sites. All other parameters were as pre-set for the software.

The data were further processed using Perseus (Version 1.5.0.9). Samples from the same cell line were processed together. Reverse and identified by site hits were removed. Proteins identified with at least 1 unique and 1 razor peptide were included for further analysis. MS/MS spectra of proteins identified by a single unique peptide are shown in Supplementary MS Spectra. Light (Bio) and heavy (K/A) intensities were logarithmized (log2). Replicates were grouped together and at least two valid values across three (SAP/TAP, THP-1, lysis buffers, formaldehyde concentrations and LidA experiments) or four (crosslinker reactivity experiment) replicates were required for at least one group as a threshold for a positive protein identification. No unique peptide threshold was applied per sample to identify as many potential interactors as possible and not exclude proteins prematurely. Missing log2 intensity values were imputed using a downshifted normal distribution (1.8 downshift, 0.3 width) for each sample individually as an estimate of the detection limit of intensity for each sample. Enrichment factors (the difference in average log2 intensity between Bio and K/A samples) were calculated using imputed values if required. Proteins were ranked according to this enrichment factor for each experiment, resulting in Top10 ranked enriched proteins. Heat maps were generated using log2 light and heavy intensities with imputed values removed. Proteins were classified into five possible categories: Bio-specific (protein is only identified in the Bio sample), Bio enriched (enrichment factor ≥2), nonspecific (-2≤enrichment factor≤2), K/A enriched (enrichment factor ≤-2) and K/A-specific (protein is only identified in the K/A sample). These 5 categories were combined into two broader groups: interactors (Bio specific and Bio enriched) and non-interacting proteins (nonspecific, K/A-enriched, and K/A-specific). MS tables are found in Supplementary MS Tables. The mass spectrometry proteomics data have been deposited to the ProteomeXchange Consortium via the PRIDE partner repository with the dataset identifier PXD003573.

##### Co-immunoprecipitation

A549-GFP-Rab2a/5c/10 cells were seeded and infected as described above. Cells were washed with 3 × 5 ml of PBS and 1 ml of Triton lysis buffer (with protease inhibitors and Benzonase) added. The cells were allowed to lyse at 4 °C for 30 min and scraped into 1.5-ml tubes. Insoluble debris was removed by centrifugation (20,000 × *g*, 15 min, 4 °C). Protein G-coupled Dynabeads (Thermo Fisher) were coated with mouse anti-GFP antibodies (Abcam, ab1218). Soluble cell lysate was cleared using uncoated protein G-coupled Dynabeads for 15 min, 4 °C. The cleared lysate was added to the coated beads and incubated for 1 h at 4 °C. The beads were washed with 1× Triton lysis buffer, 1× PBS/0.5% Triton X-100, 1× PBS/0.05% Tween20, 1 × 20 mm Tris/200 mm NaCl, and 1× PBS with 5-min incubations between each wash. Proteins on the beads were eluted by addition of 30 μl of 1× Laemmli buffer and boiling for 5 min. Proteins were separated by SDS-PAGE and transferred onto PVDF membrane for Western blot analysis. Membranes were blocked in 5% milk in PBS 0.1% Tween20. Primary antibodies (rabbit anti-GFP (1:2000, Abcam, ab290) and mouse anti-HA-HRP (1:4000, Sigma, H6533)) were added for 1 h at room temperature, the membranes washed 3 × 5 min with PBS 0.1% Tween 20 and secondary antibody (anti-rabbit IgG-HRP (1:10000, Jackson Immunoresearch, 111–035-008)) added for 1 h at room temperature if required. Western blots were visualized using EZ-ECL and a Fuji LAS3000 imager.

## Results

### 

#### 

##### The Infection-dependent Rab Binding Profile of SidM

**FIGURE 1.**
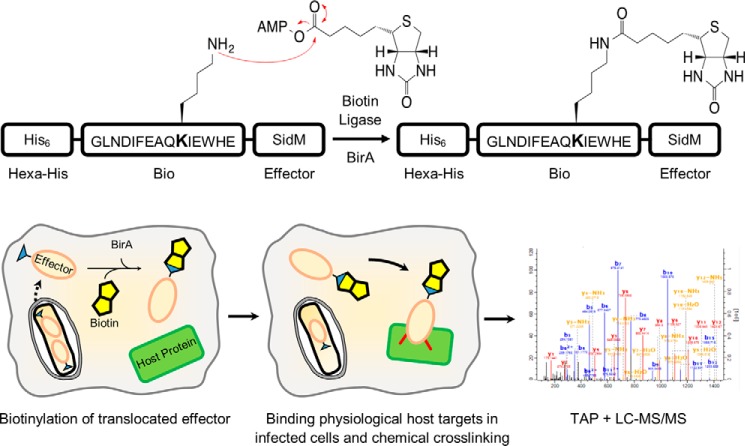
**Schematic workflow of isolation and identification of Bio-tagged effector complexes.** A549-BirA cells are infected with *Legionella* strains expressing His_6_-Bio-tagged effectors. Upon translocation, the Bio-tagged effector (*orange oval*) is biotinylated by the biotin ligase, BirA. Interactions between the effector and its host targets are stabilized using chemical crosslinking (in *red*). Subsequent tandem-affinity purification of effector complexes using His_6_/Ni^2+^ NTA followed by biotin/Neutravidin enables their composition to be determined using LC-MS/MS.

To identify the SidM binding partners during infection, A549 or THP-1 cells expressing BirA (A549-BirA and THP-1-BirA) were infected with *L. pneumophila* 130b expressing His_6_-Bio-SidM (Bio-SidM) (Fig. 1). *L. pneumophila* expressing His_6_-Bio K/A-tagged SidM (K/A-SidM), which lacks the lysine in the biotinylation site, was used as a negative control. While A549-BirA cells were infected for 24 h, due to *L. pneumophila*-induced cytotoxicity, the THP-1-BirA cells could only be infected for 6 h. Infected cells were crosslinked with 1% formaldehyde, lysed in phosphate buffer containing 1% Triton X-100 and SidM complexes were isolated either by a Neutravidin single-affinity purification (SAP) or a Ni^2+^-NTA/Neutravidin tandem-affinity purification (TAP) and analyzed by LC-MS/MS. Protein enrichment was calculated as the difference in average log2 intensity across replicates between Bio- and K/A-SidM samples. Proteins either exclusively found in the Bio-sample or had an enrichment factor ≥2 were considered SidM interaction partners. All other proteins were classified as unspecific background.

**FIGURE 2.**
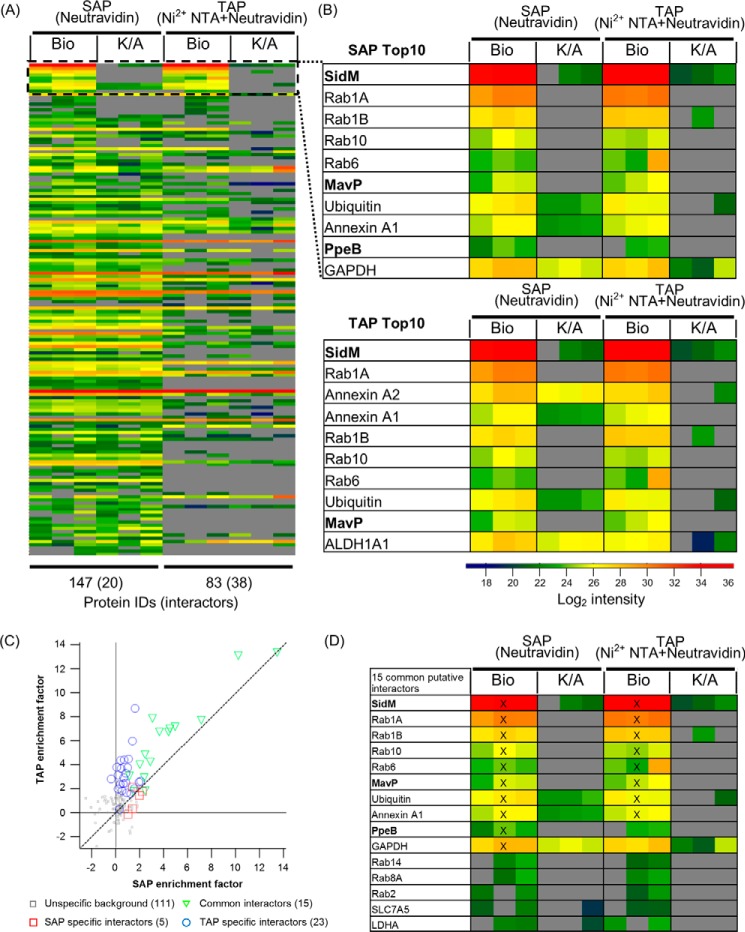
**The SidM interactome during infection.** A549-BirA cells were infected with *Legionella* expressing His_6_-Bio-SidM or His_6_-Bio K/A-SidM. A single Neutravidin purification (SAP) was compared with a tandem affinity (Ni^2+^ NTA and Neutravidin) purification (TAP). Each sample was crosslinked with 1% formaldehyde, lysed in Triton X-100, and subjected to LC/MS-MS analysis. *A*, heat map showing intensities of all identified proteins across SAP and TAP. Proteins were ranked by enrichment factors (Bio over K/A). Missing values (*gray*) were imputed as an estimate of detection limit for ranking purposes. Each column represents an individual technical replicate. *B*, zoom of Top 10-enriched proteins identified in SidM complexes for SAP and TAP. *Legionella* proteins are in *bold. C*, plot of average protein enrichment factors of SAP replicates against TAP samples. Common interactors are shown as *green triangles*. SAP-specific and TAP-specific interactors are in *red squares* and *blue circles*, respectively. *D*, heat map showing the 15 common interactors. Top 10-enriched proteins are marked with a *cross* (X).

The bait protein SidM was identified in Bio-SidM samples from both A549 and THP-1 cells. However, while SidM was detected with the highest log2 intensity of 34 in A549 Bio-SidM samples (both SAP and TAP) (Fig. 2, *A* and *B*) and was the most enriched protein (enrichment factor of 13) (Fig. 2*C*), its intensity from THP-1 cells was ∼30-fold lower and its enrichment factor 3 units lower (Supplemental Fig. S1 and Supplementary MS tables). In addition to lower SidM intensities, the THP-1 samples yielded fewer proteins which were classified as interactors (Supplemental Fig. S1*A*). As THP-1 samples approached the limit of detection and A549 samples provided more analyzable data, all subsequent experiments were performed using A549-BirA cells. However, although not many interactors were detected for THP-1 samples, Rab1A was found under both SAP and TAP conditions (Supplemental Fig. 1*B*).

In A549-BirA samples, SAP identified 147 proteins, although only 20 of these (13.6%) passed the selection criteria as interaction partners. Of the 83 TAP identified proteins, 38 were classed as interaction partners (45.8%); 15 of these interactors were shared between SAP and TAP (Fig. 2*C*). Enrichment factors of these common interactors were higher in TAP conditions than SAP conditions. Importantly, eight common targets were found in the Top10 ranked enriched proteins for both SAP and TAP (Fig. 2*D*). Multiple Rab GTPases, including 1A, 1B, 6, and 10, were detected within the Top10 ranked enriched proteins under both SAP and TAP (Fig. 2*B*), while Rab2, 8A, and 14 were ranked lower. Detection of the known physiologically relevant SidM binding partners Rab1A and 1B validated the BirA/Bio-tag system. In addition to the Rab GTPases, annexin A1 and ubiquitin were consistently found among the Top10 SidM targets.

Along with host proteins, the T4SS *Legionella* effectors Lpw_31531 (MavP), Lpw_17241 (PpeB), and Lpw_25181 (Lpg2327) were identified as interactors of SidM (Fig. 2*B* and Supplementary MS Tables). MavP and PpeB were ranked within the Top10 for SAP while only MavP was a Top10 hit for TAP. As TAP reduced unspecific background binders while facilitating identification of interactors, it was used for all subsequent experiments.

##### Chaotropic Agents Increase Complexity of the Isolated Proteome but Not SidM-specific Interactome Coverage

To determine the effects of different lysis conditions on proteome solubilization and protein identification, we compared lysis buffers containing either 1% Triton X-100 and 6 m guanidinium chloride (GnCl/Triton) or buffers with 1% Triton X-100 (Triton), 1% 3-[(3-cholamidopropyl)dimethylammonio]-1-propanesulfonate (CHAPS), or 0.5% sodium dodecyl sulfate (SDS). All samples were crosslinked with 1% formaldehyde before lysis and complexes isolated by TAP.

**FIGURE 3.**
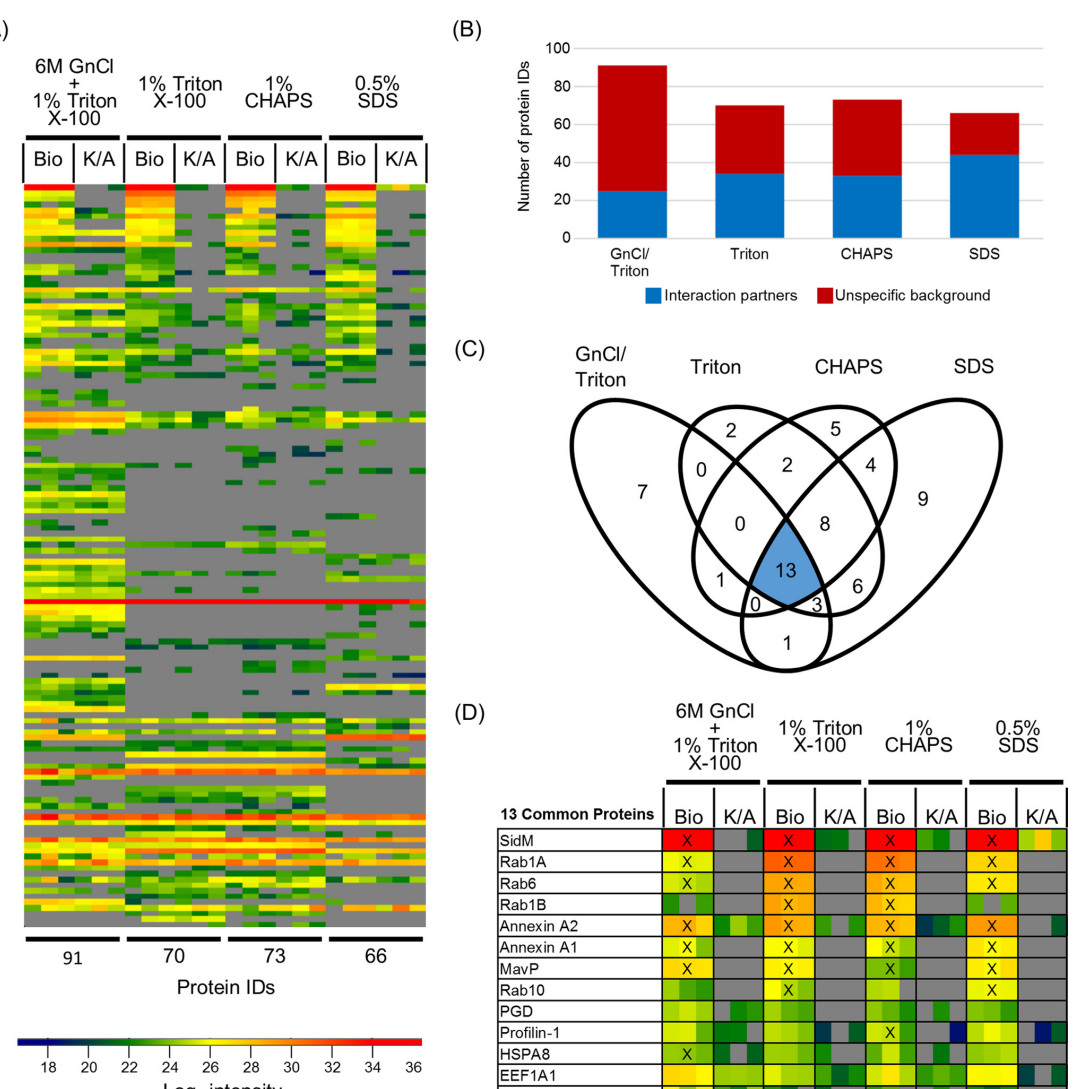
**The effect of lysis conditions on the efficiency of SidM effector complex isolation.** *A*, heat map of all identified proteins across all lysis conditions ranked based on the Triton X-100 lysis condition enrichment factors. *B*, comparison of the breakdown of the identified proteins into interactors and unspecific background. *C*, Venn diagram showing the overlap of SidM interactors identified between experimental conditions. *D*, heat map showing the log2 intensities of the 13 interactors found across all four lysis conditions, ranked according to the Triton condition. Proteins found within the Top10-enriched proteins for each individual condition are indicated by a *cross* (X).

Use of GnCl/Triton buffer enabled identification of 91 proteins relative to 70, 73, and 66 IDs from Triton, CHAPS and SDS treated samples, respectively (Fig. 3*A*). Although GnCl/Triton treatment resulted in more protein identifications, most of these IDs were unspecific background and provided the fewest interaction partners, 25, out of all four conditions (Fig. 3*B*). Triton, CHAPS, and SDS identified 34, 33, and 44 interactors, respectively.

Altogether, the four lysis buffer conditions revealed 61 interactors, of which 13 were identified in all four conditions (Fig. 3*C*). Only Rab1A, Rab6, annexin A1, annexin A2, and MavP were consistently identified as Top10 interactors across all conditions (Fig. 3*D*). Notably, although Rab1A was identified and ranked within the Top10 under protein denaturing conditions, its intensity was 40/25-fold (GnCl/Triton) and 12/7-fold (SDS) lower than those found in non-denaturing (Triton and CHAPS) conditions, respectively, suggesting that denaturing conditions adversely affect the stability of the Rab1A-SidM interaction. Taken together, although chaotropic agents aid in proteome solubilization, harsher lysis conditions seem to decrease confidence in specific interactor identification. Triton X-100 was therefore used for subsequent experiments.

##### Chemical Crosslinking Increases Interactome Coverage and Complexity

To determine if additional interaction partners may be identified through increased crosslinking, two formaldehyde concentrations, 1 and 3%, were applied; 0% formaldehyde was used as a control.

**FIGURE 4.**
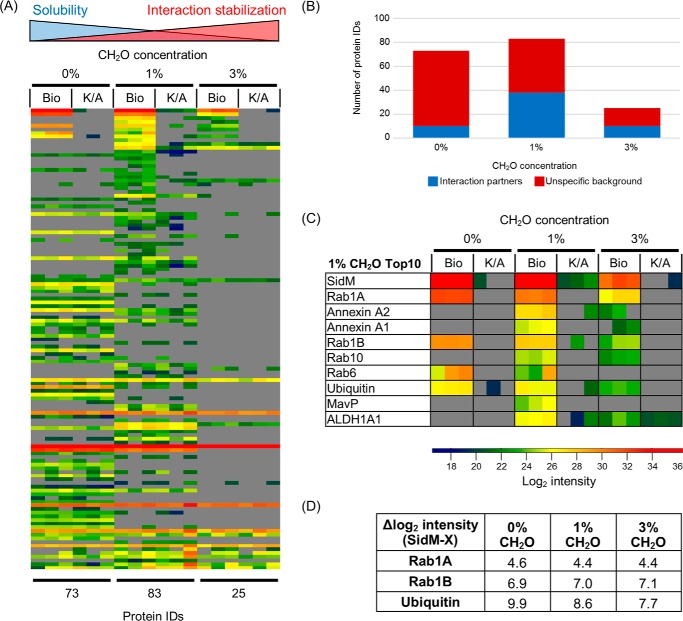
**The effect of formaldehyde crosslinking concentration on SidM interactomes.** *A*, heat map of all identified proteins across the three concentrations ranked based on enrichment factors from the 1% formaldehyde experimental condition. *B*, breakdown of the identified proteins into interactors and unspecific background. *C*, zoom of Top 10-enriched proteins identified in SidM complexes isolated from 1% formaldehyde crosslinking. *D*, table showing the difference in average log2 intensity between SidM and three proteins (Rab1A, Rab1B, and ubiquitin) found across all three experimental conditions.

MS analysis identified 73, 83, and 25 proteins in the 0%, 1%, and 3% formaldehyde-treated samples, respectively (Fig. 4*A*). Within these 10, 38 and 10 proteins were classified as interactors, respectively (Fig. 4*B*). The intensity of SidM found after 3% formaldehyde treatment was 36-fold and 11-fold lower than those found at 0 and 1%, respectively (Fig. 4*C*). Although the intensity of SidM was 3-fold lower with 1% formaldehyde compared with no crosslinking (0%), the number of interaction partner IDs was ∼4-fold higher (38 *versus* 10 IDs) with the crosslinker. Rab1A, Rab1B and ubiquitin were identified under all conditions tested (Fig. 4*C*). The intensities of Rab1A and Rab1B were proportional to the intensity of SidM isolated from each sample (Fig. 4*D*). In contrast, the relative intensity of ubiquitin increased with formaldehyde concentration. Five proteins within the Top10 ranked enriched proteins according to 1% formaldehyde were only identified in the presence of crosslinking: annexin A2, annexin A1, Rab10, MavP, and ALDH1A1 (Fig. 4*C*). These proteins except MavP were also identified under 3% formaldehyde conditions. In summary, these results suggest that moderate chemical crosslinking with 1% formaldehyde allows the recovery of more complex effector interactomes.

##### Crosslinker Reactivity Influences the SidM Interactome

We next tested if using crosslinkers with different amino acid reactivity and linker length (Supplemental Table S6) changed the interactome of SidM. Dithiobis(succinimidyl propionate) (DSP), dithiobismaleimidoethane (DTME), and succinimidyl 4-(*N*-maleimidomethyl)-cyclohexane-1-carboxylate (SMCC) were employed. A combination of DSP and DTME was included to determine whether synergistic effects can be achieved. These four conditions were compared with no crosslinking and 1% formaldehyde conditions.

**FIGURE 5.**
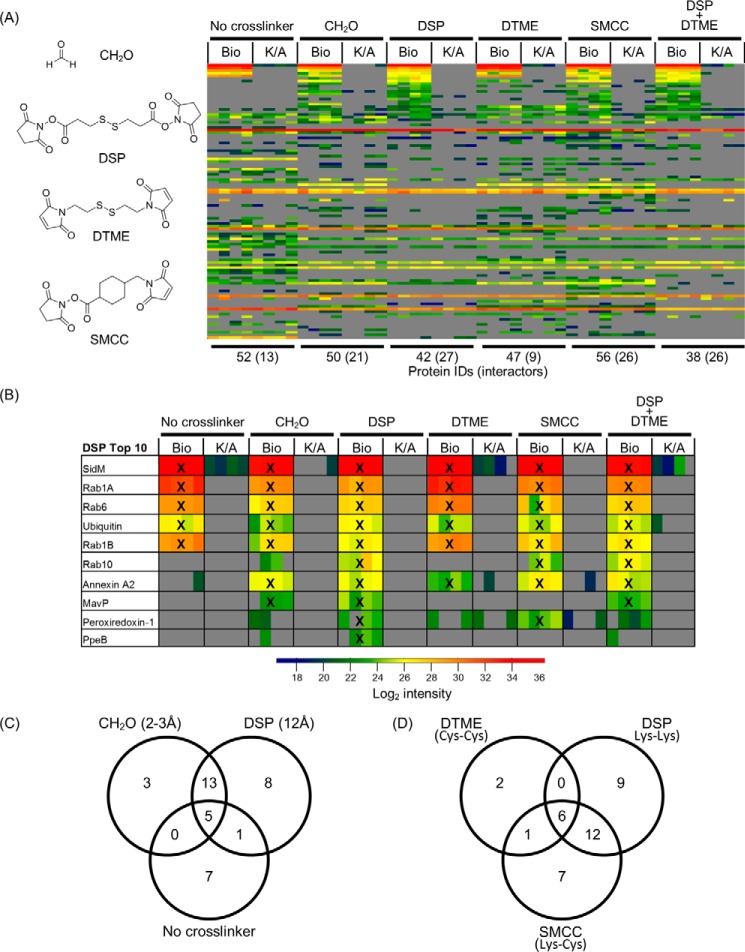
**The effect of crosslinker length and reactivity on SidM interactomes.** Six crosslinking conditions were compared: no crosslinker, 1% formaldehyde, 1 mm DSP, 0.5 mm DTME, 1 mm SMCC, and 1 mm DSP/0.5 mm DTME. *A*, heat map of all identified proteins across all crosslinking conditions ranked based on enrichment factors calculated from the DSP condition. *B*, heat map showing the Top10 enriched proteins identified in SidM complexes found under DSP crosslinking conditions. Proteins also found ranked in the Top10 of their respective crosslinking condition are marked with a *cross* (X). *C*, Venn diagram comparing interactors identified by varying lysine-lysine crosslinking length. *D*, Venn diagram comparing SidM interactors identified by varying crosslinker reactivity.

All six conditions yielded similar numbers of protein IDs (∼50) (Fig. 5*A*). However, the number of interactors ranged from 13 (no crosslinking) and 9 (DTME) up to 27 under DSP conditions. Formaldehyde revealed 21 interactors while SMCC and DSP+DTME both identified 26. Comparison of the identities of these interactors revealed that the five common proteins (SidM, Rab1A, Rab1B, Rab6, and ubiquitin) were all ranked within the Top10-enriched proteins across all conditions (Fig. 5*B*). Formaldehyde and SMCC shared a further two and three Top10 targets with DSP, respectively. DTME treatment resulted in the identification of one additional shared Top10 protein with DSP but otherwise resembled the uncrosslinked sample (Fig. 5*B*). The Top10 profile of the DSP+DTME condition overlapped in eight Top10 hits with the DSP only condition, suggesting that it is predominantly dictated by DSP crosslinking. Although the Top10 ranked interactors were relatively similar across most conditions, there were differences in identification of lower ranked putative interactors dependent on both crosslinking length (Fig. 5*C*) and crosslinking reactivity (Fig. 5*D*).

Detection of the Top10 targets Rab10, MavP and annexin A2 showed clear crosslinker reactivity dependence. Only in the presence of a crosslinker with at least one amine reactive group was Rab10 identified (formaldehyde, DSP, SMCC, and DSP+DTME) (Fig. 5*B*). The MavP/SidM interaction was only identified upon addition of formaldehyde or DSP (Lys-Lys crosslinkers) while the interaction between SidM and annexin A2 just required the presence of a crosslinker, independent of reactivity.

**FIGURE 6.**
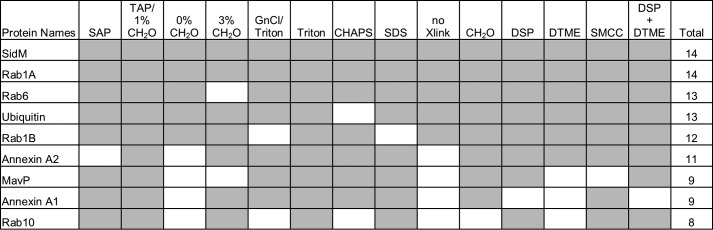
**SidM interacting partners.** Table of SidM interactors found as a Top10 hit in at least 50% of all experimental conditions tested. *Gray boxes* indicate a Top10 hit.

In summary, we found that variation of crosslinking and lysis conditions influences the coverage of the isolated proteomes; however across all the conditions the identified highest confidence (Top10 hit in at least 50% of all experimental conditions tested) interactors of SidM remain very consistent (Fig. 6). Importantly, the data suggests that ubiquitin, annexin A1, annexin A2, MavP are putative novel interaction partners and that Rab1A, Rab1B, Rab6, and Rab10 are the predominant Rab GTPases targeted by SidM during infection.

##### The Host Cell Interactome of LidA

**FIGURE 7.**
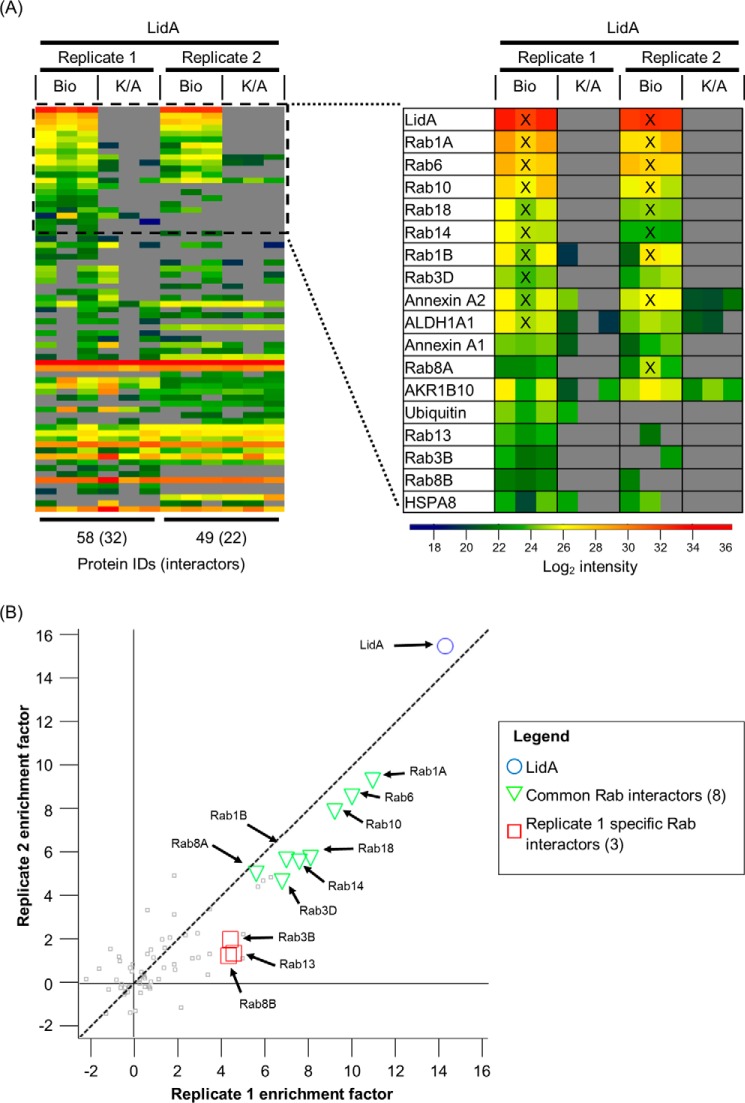
**The LidA interactome during infection.** Two biological experiments (replicate 1 and 2) were performed in technical triplicate. *A*, heat map of all identified proteins ranked based on the enrichment factor (Bio over K/A) of replicate 1. Top 10 ranked hits are indicated with a *cross* (X) *B*, plot of average protein enrichment factors between two biological LidA experiments. LidA is highlighted as a *blue circle*. Rab GTPases identified as interactors found in both experiments are shown as *green triangles*. Interacting Rab GTPases identified in only replicate 1 are depicted by *red squares*.

As the SidM interactome data revealed a more specific Rab GTPase binding profile during infection compared with *in vitro* studies, we similarly investigated the *in vivo* interactome of the other promiscuous Rab GTPase binding effector LidA. A549-BirA cells infected with *L. pneumophila* 130b expressing His_6_-Bio-tagged-LidA were subjected to 1% formaldehyde crosslinking, Triton X-100 lysis, and TAP. The top interactors of LidA were predominantly Rab GTPases. Rab1A, 1B, 3D, 6, 8A, 10, 14, and 18 were consistently identified across both experiments, whereas Rab3B, 8B, and 13 were only identified robustly in one experiment (Fig. 7*A*). Although absolute enrichment factors differed between biological replicates, ranking of the LidA-interacting Rab GTPases was consistent (Fig. 7*B*). Annexin A2 was found as the only non-Rab GTPase Top10 hit across both experiments. While Rab14 and 18 were Top10 ranked specifically for LidA, the data shows that Rab1A, 1B, 6, and 10 are targeted by both effectors SidM and LidA.

##### Rab10 Interacts with SidM and LidA during Infection

To validate the interaction of SidM and LidA with the identified Rab GTPases during infection, we performed co-immunoprecipitations of the effectors with Rab2A, Rab5C, and Rab10. While Rab10 was a Top10 target for both SidM and LidA, Rab2A was only found outside the Top10 hits in SidM interactomes and Rab5C was never identified in either effector interactome. A549 cells stably expressing either GFP-Rab2A, -Rab5C, or -Rab10 were infected with *L. pneumophila* strains expressing either 4HA-tagged SidM, LidA, or the negative control PI4P-binding domain of the effector SidC, which localizes to the LCV but has no reported Rab GTPase binding partners.

**FIGURE 8.**
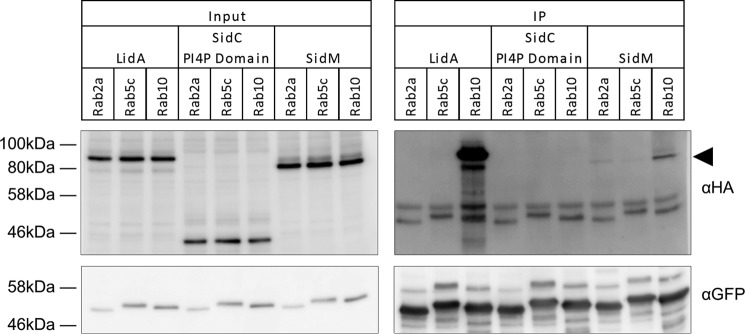
**SidM and LidA co-immunoprecipitate with Rab10.** GFP-Rab transduced A549 cells (Rab2a, Rab5c, and Rab10) were infected with *Legionella* strains expressing HA-tagged LidA, SidC (PI4P binding domain) or SidM. GFP-Rab GTPases were immunoprecipitated using anti-GFP beads. Immunoprecipitated proteins were analyzed by Western blot using anti-GFP and anti-HA antibodies. The SidM band is indicated by the *black arrowhead*.

LidA was specifically co-immunoprecipitated with Rab10, while SidM was co-immunoprecipitated with Rab10 and to a lesser extent with Rab2A (Fig. 8). Neither effector was co-immunoprecipitated with Rab5C. The PI4P-binding domain of SidC was not precipitated with any of the three Rab GTPases. Taken together, this strengthens the conclusion that Rab10 is targeted by both SidM and LidA during infection and reflects the Rab GTPase enrichment ranking obtained from SidM interactomes.

## Discussion

In this work we studied the interactomes of SidM and LidA during infection. Preliminary optimization studies revealed that due to *L. pneumophila*-induced cytotoxicity (even at MOI of 1) and low bait protein yield, THP-1 cells were not suitable host cells for this analysis. In contrast, A549 cells allowed us to define the infection-dependent Rab GTPase binding profiles of SidM and LidA. Rab1A and Rab1B were confirmed as strong interactors of both effectors. Rab6 and Rab10 were found as additional Rab GTPase targets for SidM with consistently high enrichment factors. Although Rab1A, 1B, and 6 were identified in the absence of crosslinking, Rab10 was only discovered with crosslinkers with at least one lysine reactive moiety. As the only two cysteines in SidM are buried inside its structure (PBD ID: 3NKU, 3JZA) (10, 25), DTME and DSP+DTME crosslinking did not provide an enhanced interactome above no crosslinking or DSP only samples respectively. Formaldehyde crosslinking did not enhance the interactions of Rab1A and Rab1B with SidM. This suggests that not all protein-protein interactions are amenable to crosslinking and could explain why the transmembrane SidM partners syntaxins were not detected in this screen (26). Photoactivatable radical-based crosslinkers could be used to broaden the range of detectable protein-protein interactions. Furthermore, the use of crosslinkers permitting the identification of the crosslinking sites could provide information on the binding interfaces. The observation that all four high confidence SidM-interacting Rab GTPases were detected under both SAP and TAP (1% formaldehyde, Triton X-100 lysis) conditions suggests that TAP reduced background proteins while also maintaining key physiologically relevant interactions of effectors during infection.

**FIGURE 9.**
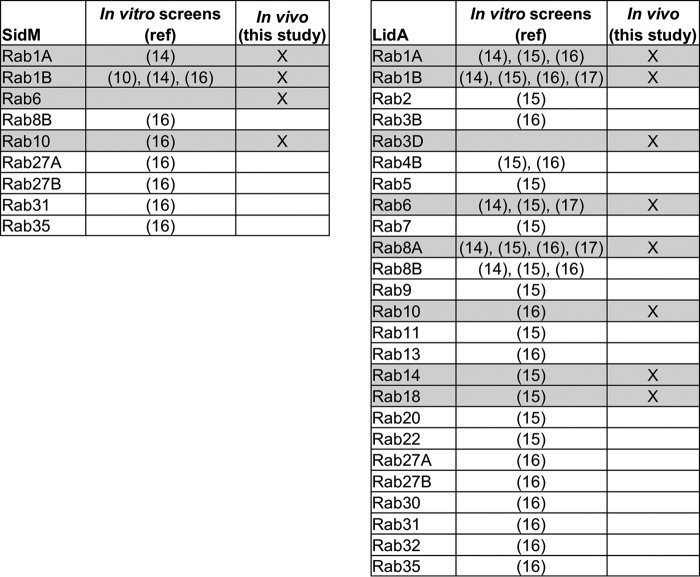
**SidM and LidA show a more restricted infection-dependent Rab GTPase binding profile than *in vitro* assays.** Comparison of the *in vitro* Rab GTPase targets of SidM and LidA identified in previous studies and the *in vivo* Rab partners found in this study. Rab GTPases identified as interactors in this study are highlighted in *gray*.

Although SidM and LidA share Rab GTPase binding partners, both effectors were never found in the same interactome, suggesting that SidM and LidA act independently and mutually exclusively on their targets. Whether *Legionella* controls this through temporal regulation of translocation or spatial separation in LCV micro-domains is yet to be determined. In addition to Rab1A, 1B, 6, and 10 which LidA and SidM both bind, LidA also bound Rab3B, 3D, 8A, 8B, 13, 14, and 18. These differences in Rab GTPase binding profiles in addition to not all reported LCV-bound Rab GTPases being found in either effector interactome suggest that the identified interactomes are not simply a result of proximity crosslinking of effectors and proteins which are present on the LCV simultaneously. This is supported by the observation that the LCV-bound SidC PI4P binding domain does not co-immunoprecipitate with Rab10. In contrast, the co-immunoprecipitation of SidM and LidA with Rab10 supports the result that Rab10 is a genuine target of both effectors during infection. Although LidA binding to multiple Rab GTPases has been well studied *in vitro*, only its interactions with Rab1 and Rab6 have been previously characterized in the context of infection (14, 27). LidA interacts *in vitro* with similar high affinity with Rab1 and Rab8 while it binds Rab6 with slightly weaker affinity albeit all in the picomolar range (17). Interestingly, Rab1A and Rab6 were consistently the two most enriched interactors in LidA complexes but Rab8A was ranked lower. Additionally, Rab10, 14, and 18, whose interaction with LidA has not been studied in detail yet, were ranked higher than Rab8A consistently. This suggests that more complex parameters than just the binding affinities govern the Rab-binding preferences of LidA during infection and that caution should be employed when extrapolating *in vitro* binding data to infection. Using the BirA/Bio-tag system, we have further confined the promiscuous Rab GTPase binding capabilities of SidM and LidA than in *in vitro* assays, thereby revealing a more stringent subset of Rab GTPases which these two effectors bind during infection (Fig. 9).

Multiple Rab GTPases are known to play key roles during *Legionella* infection. Rab1A is involved in the recruitment of ER-derived vesicles to the LCV while Rab6 and Rab10 promote intracellular replication, although the mechanisms remain unknown (7, 27). In contrast, Rab14 has an adverse effect on *Legionella* intracellular replication (7). Interestingly, this contrasts with the important role Rab14 has for the efficient intracellular growth of *Salmonella* (28) and *Chlamydia* (29). In addition, Rab14 has also been implicated in lipid manipulation on the LCV (7). Rab18 has not been associated with bacterial pathogenesis but has been shown to be important for ER structure (30).

In addition to host cell proteins, SidM interactomes also contained some *Legionella* proteins. Notably, only known *Legionella* effectors, but not housekeeping proteins, were identified in interactomes showing the strength of the method to specifically isolate translocated effector complexes. The data suggest that MavP might be a SidM-interacting partner. Indeed a few effector-effector interactions (*e.g.* LubX-SidH, SidJ-SidE) have already been reported (31, 32). LubX-SidH and SidJ-SidE interactions seem to represent regulatory relationships; where one effector controls the half-life of the other in the cell. The functional consequences of SidM-MavP interaction during *Legionella* infection requires further investigation.

In conclusion, in this study we used a refined TAP approach and semi-quantitative proteomics to define a subset of Rab GTPases that interact with SidM and LidA during infection. While several *in vitro* screens revealed that SidM binds up to 9 Rab GTPases and LidA binds 25 Rab GTPases, we have shown that intracellular SidM specifically binds Rab1A, 1B, 6, and 10 during infection while LidA binds Rab1A, 1B, 3D, 6, 8A, 10, 14, and 18.

## Author Contributions

E. C. S. designed and performed the majority of the experiments, analyzed the data, and wrote the paper. G. N. S. co-supervised the project, performed experiments and wrote the paper. D. C. and C. M. performed experiments. A. M. conceived the study and wrote the paper. M. B. provided technical assistance and wrote the paper. E. W. T. and G. F. supervised the project and wrote the paper. All authors reviewed the results and approved the final version of the manuscript.

## Supplementary Material

Supplemental Data

## Footnotes

4 The abbreviations used are:

LCV: *Legionella* containing vacuole
T4SS: type IV secretion system
ER: endoplasmic reticulum
PI4P: phosphatidylinositol 4-phosphate
TAP: tandem-affinity purification
LC-MS/MS: liquid chromatography tandem mass spectrometry
SAP: single-affinity purification
MS: mass spectrometry
ID: identification.
