# Supplementary material for: The Rab-binding Profiles of Bacterial Virulence Factors during Infection
Source: J Biol Chem. 2016 Jan 11;291(11):5832–43. doi: 10.1074/jbc.M115.700930 (PMC4786718; doi:10.1074/jbc.M115.700930)
Supplement: Supplemental Data [file supp_291_11_5832__index.html]

The Rab-binding profiles of bacterial virulence factors during infection — The Rab-binding Profiles of Bacterial Virulence Factors during Infection — The Rab-binding Profiles of Legionella Effectors — Supplemental Data 

# The Rab-binding Profiles of Bacterial Virulence Factors during Infection

## Supplemental Data

- Supplementary MS spectra (.pdf, 1.6 MB) - Supplementary MS spectra
- Supplementary information (.docx, 122 KB) - Supplementary information
- Supplementary MS tables (.xlsx, 973 KB) - Supplementary MS tables
