# Supplementary material for: The Rab-binding Profiles of Bacterial Virulence Factors during Infection
Source: J Biol Chem. 2016 Jan 11;291(11):5832–43. doi: 10.1074/jbc.M115.700930 (PMC4786718; doi:10.1074/jbc.M115.700930)

Raw file	Scan	Method	Score	m/z	Gene names
ECS_3B	24134	FTMS; HCD	98.04	573.35	ARF5;ARF1;ARF3

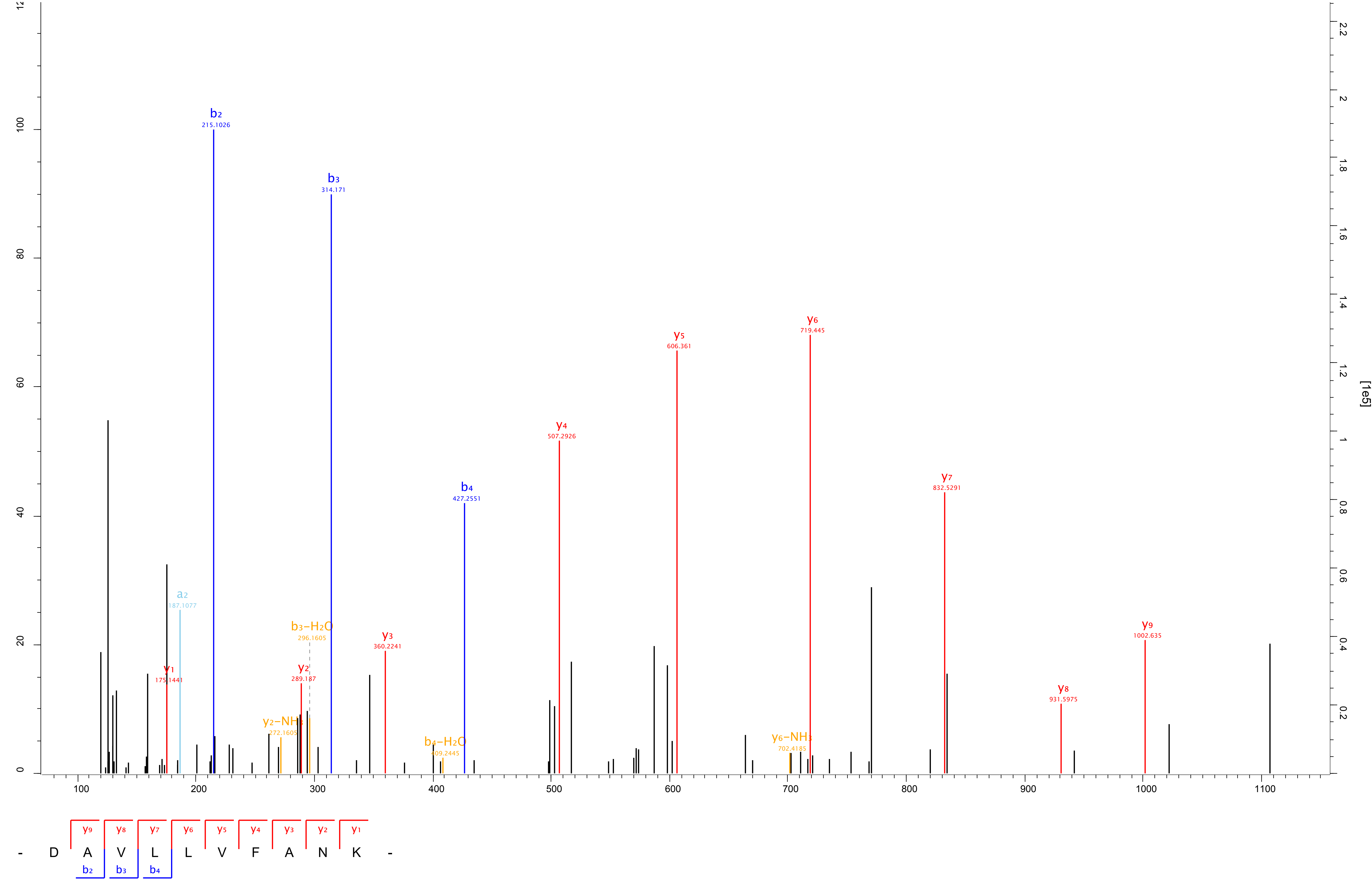

Raw file	Scan	Method	Score	m/z	
ECS_D6	8866	FTMS; HCD	43.68	456.28	CON__ENSEMBL:ENSBTAP00000038253

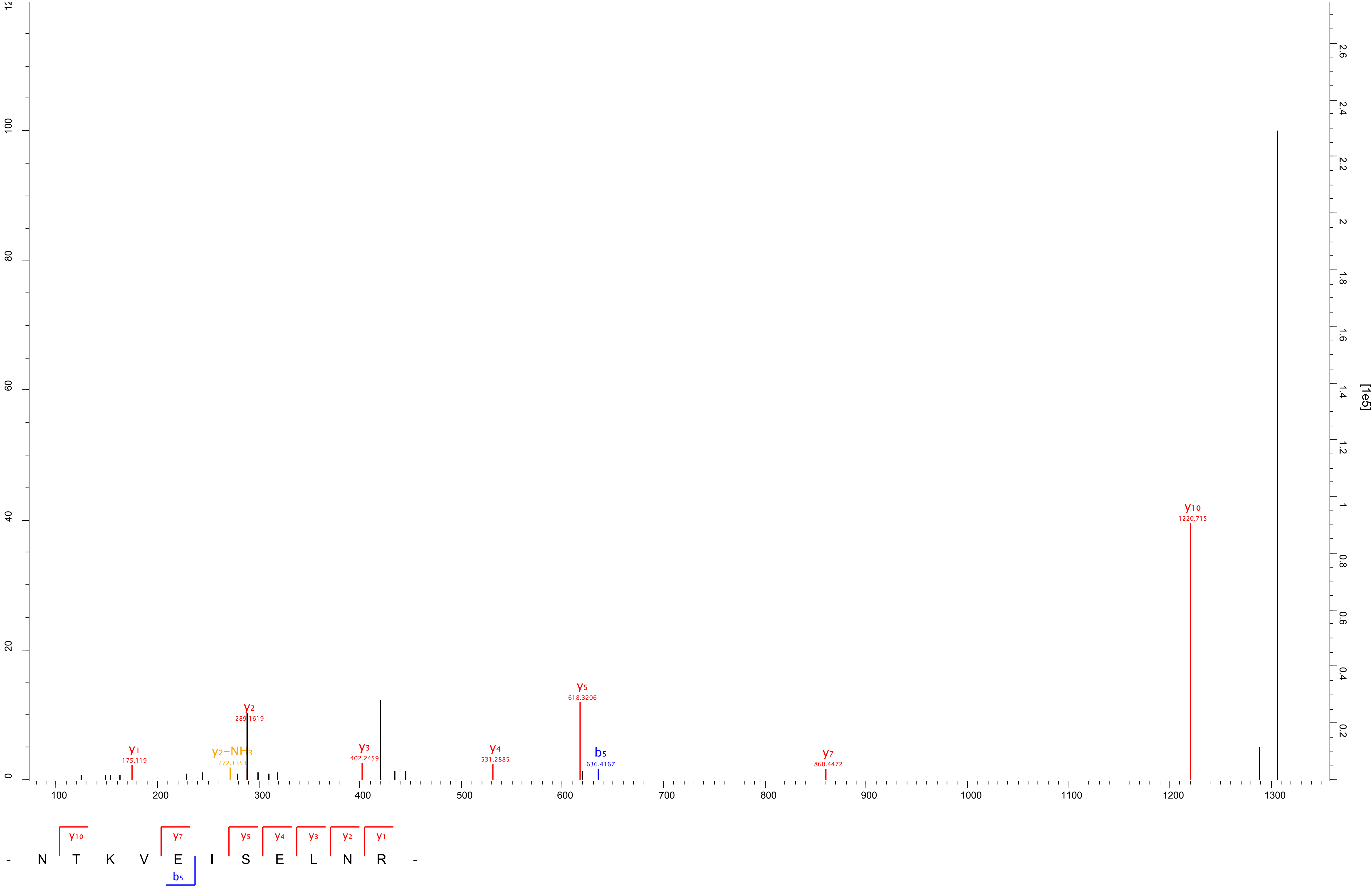

Raw file	Scan	Method	Score	m/z	
ECS_D5	4284	FTMS; HCD	65.25	405.71	CON__P04259

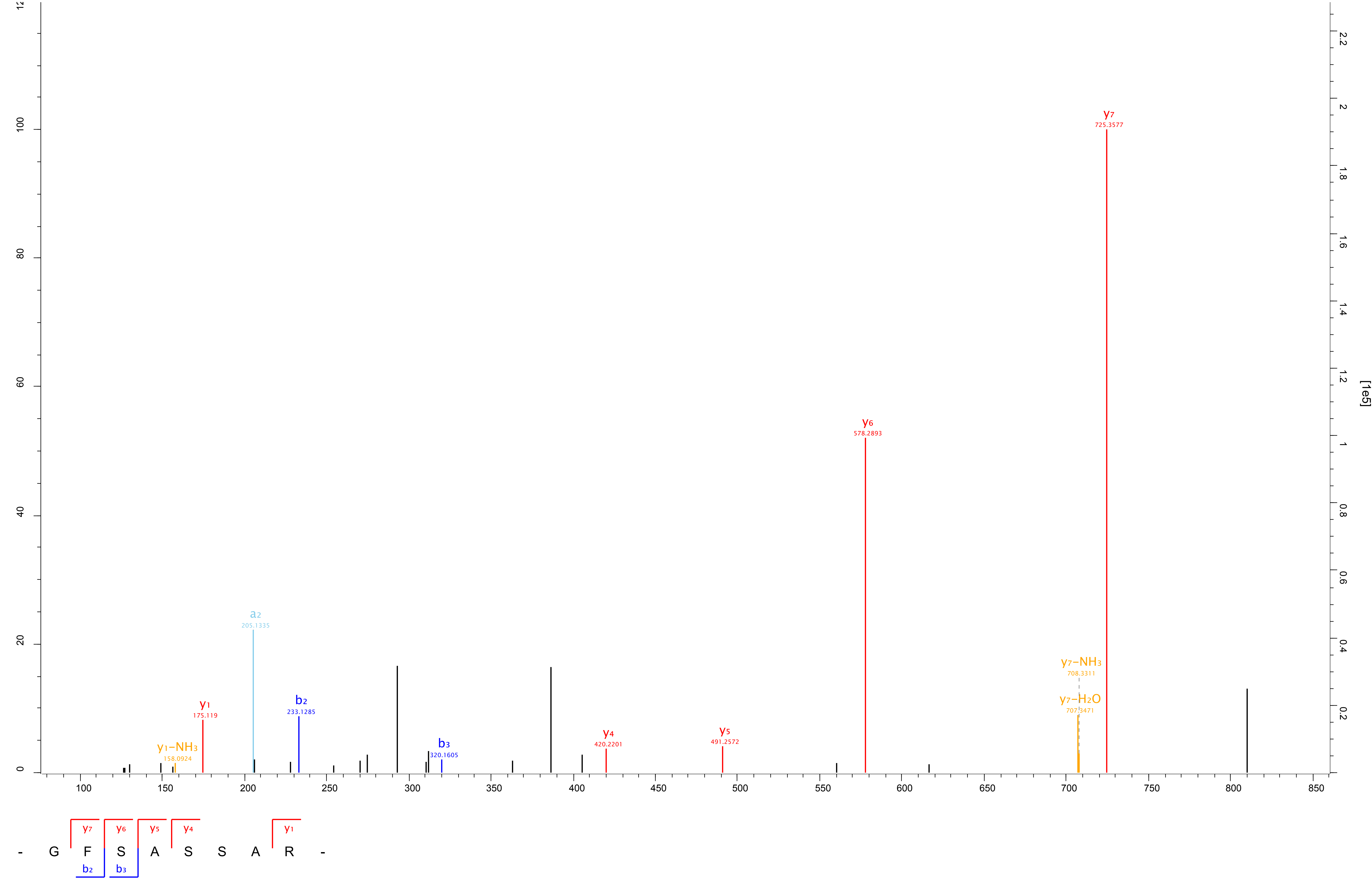

Raw file	Scan	Method	Score	m/z	
ECS_C2	13378	FTMS; HCD	78.9	638.36	CON__REFSEQ:XP_986630

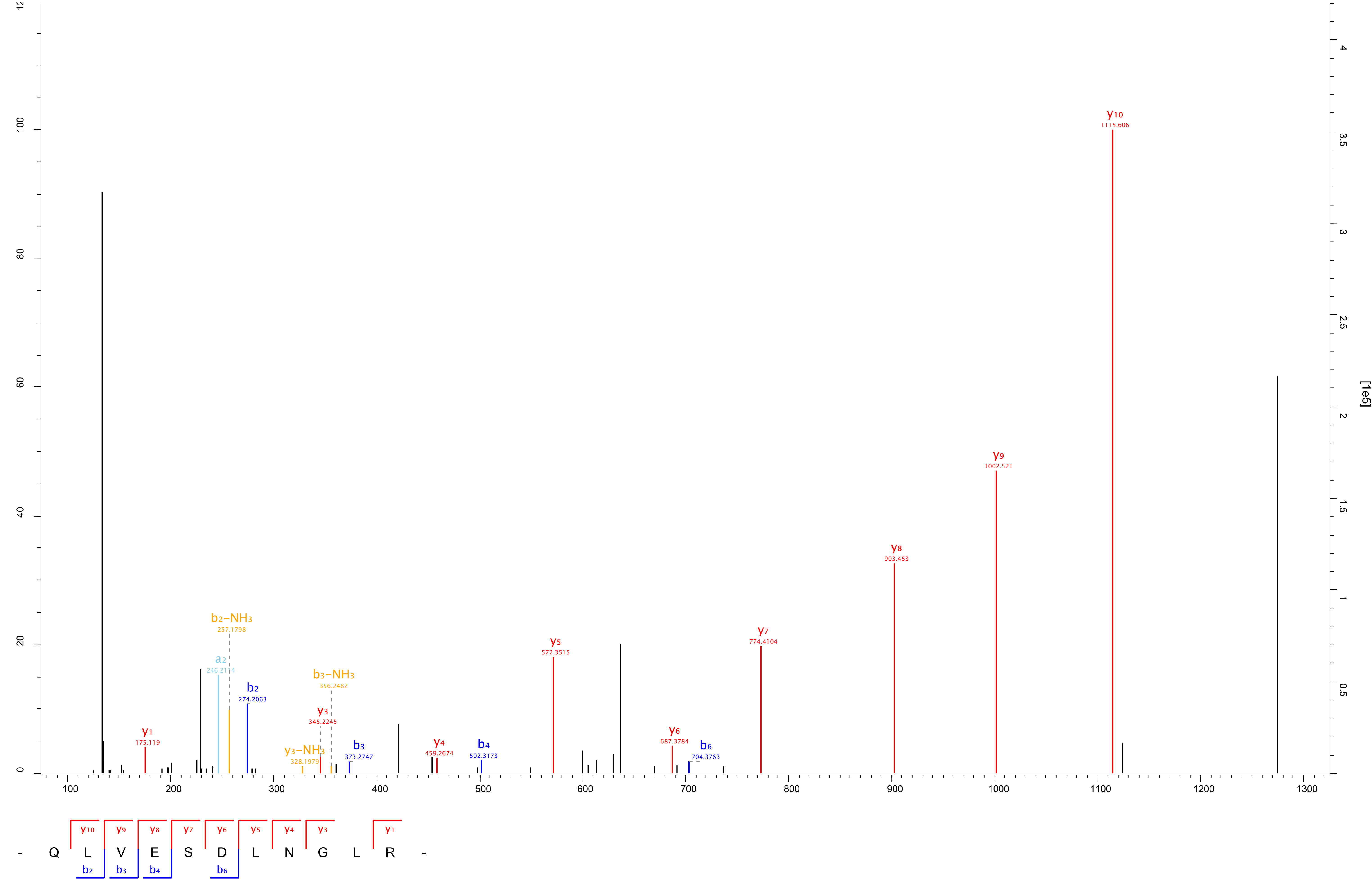

Raw file	Scan	Method	Score	m/z	Gene names
ECS_A12	17329	FTMS; HCD	94.47	616.87	RAB6A;RAB6B

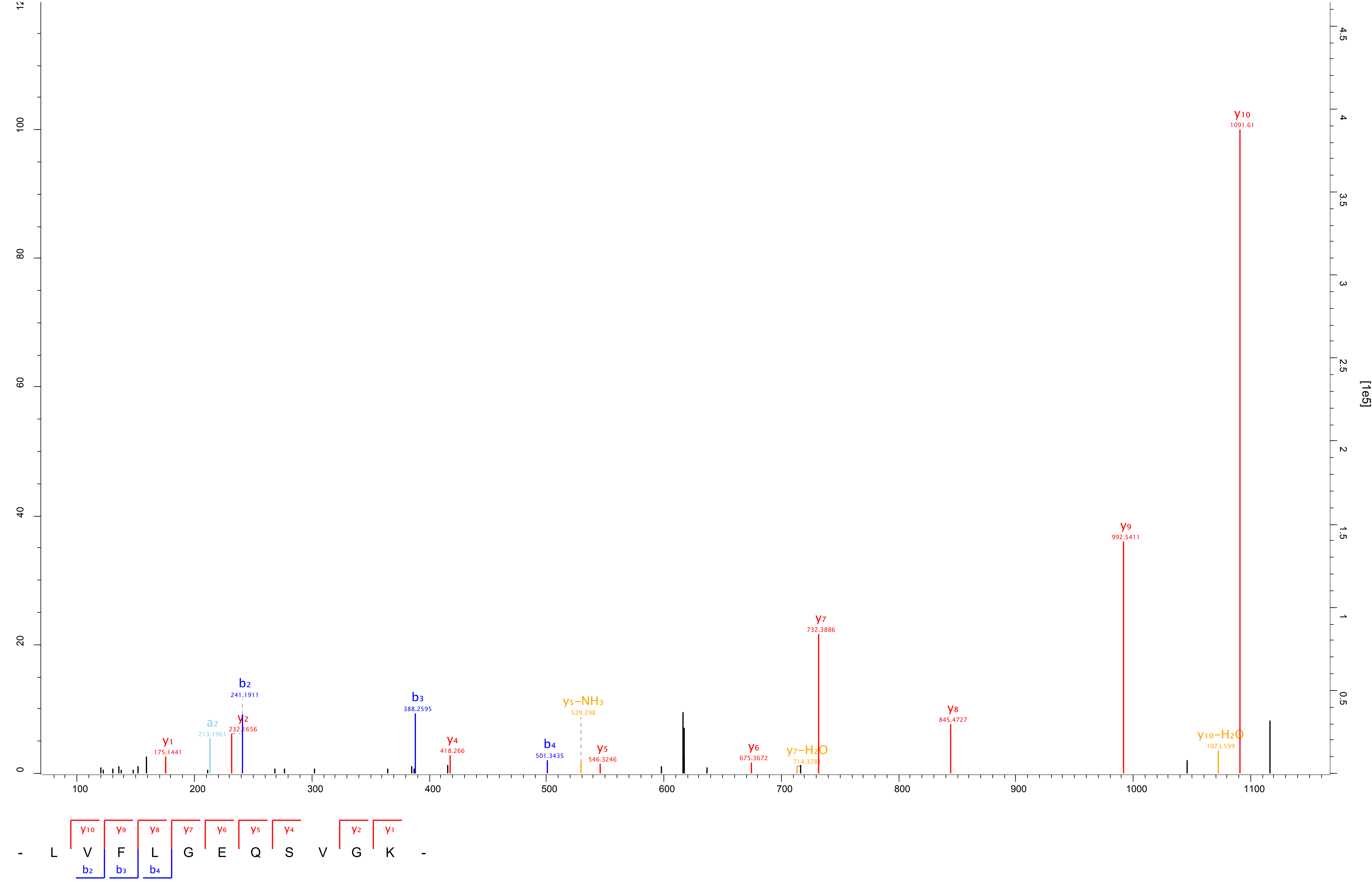

Raw file	Scan	Method	Score	m/z	Gene names
ECS_C2	13669	FTMS; HCD	115.71	539.36	KRT36

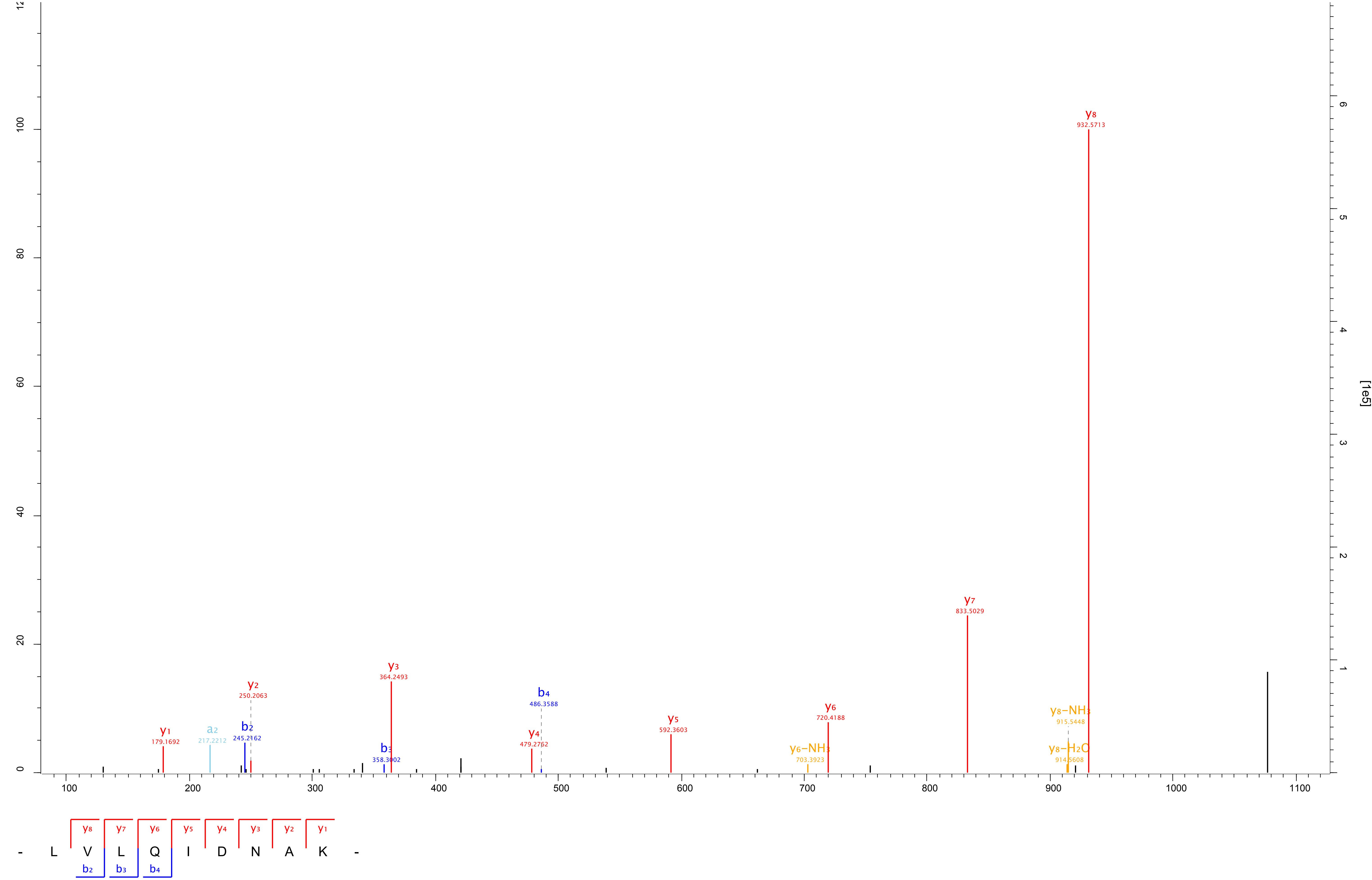

Raw file	Scan	Method	Score	m/z	Gene names
ECS_A6	13295	FTMS; HCD	82.29	362.92	SERPINB3

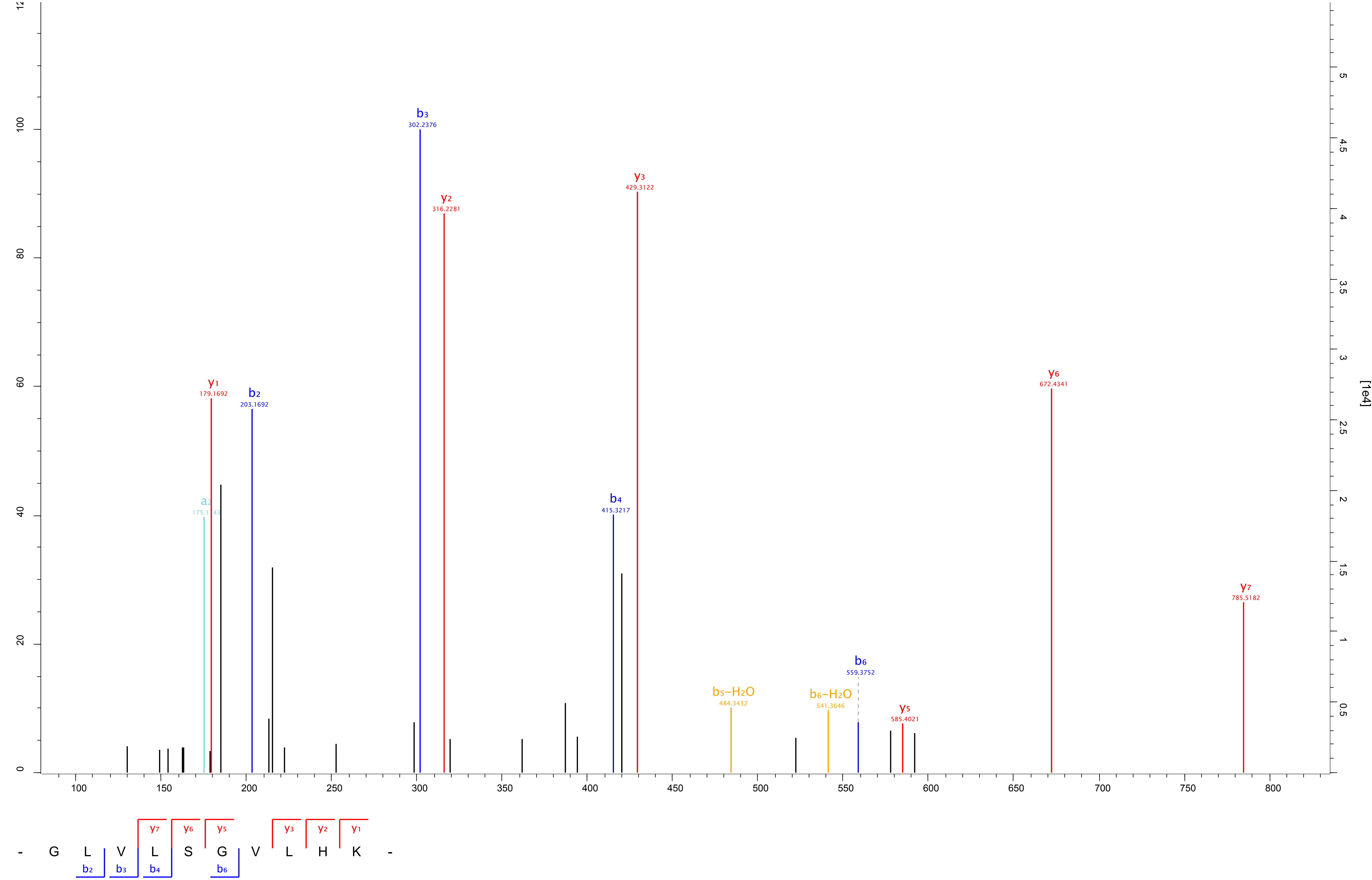

Raw file	Scan	Method	Score	m/z	Gene names
ECS_4B	20360	FTMS; HCD	214.16	810.02	KRT2

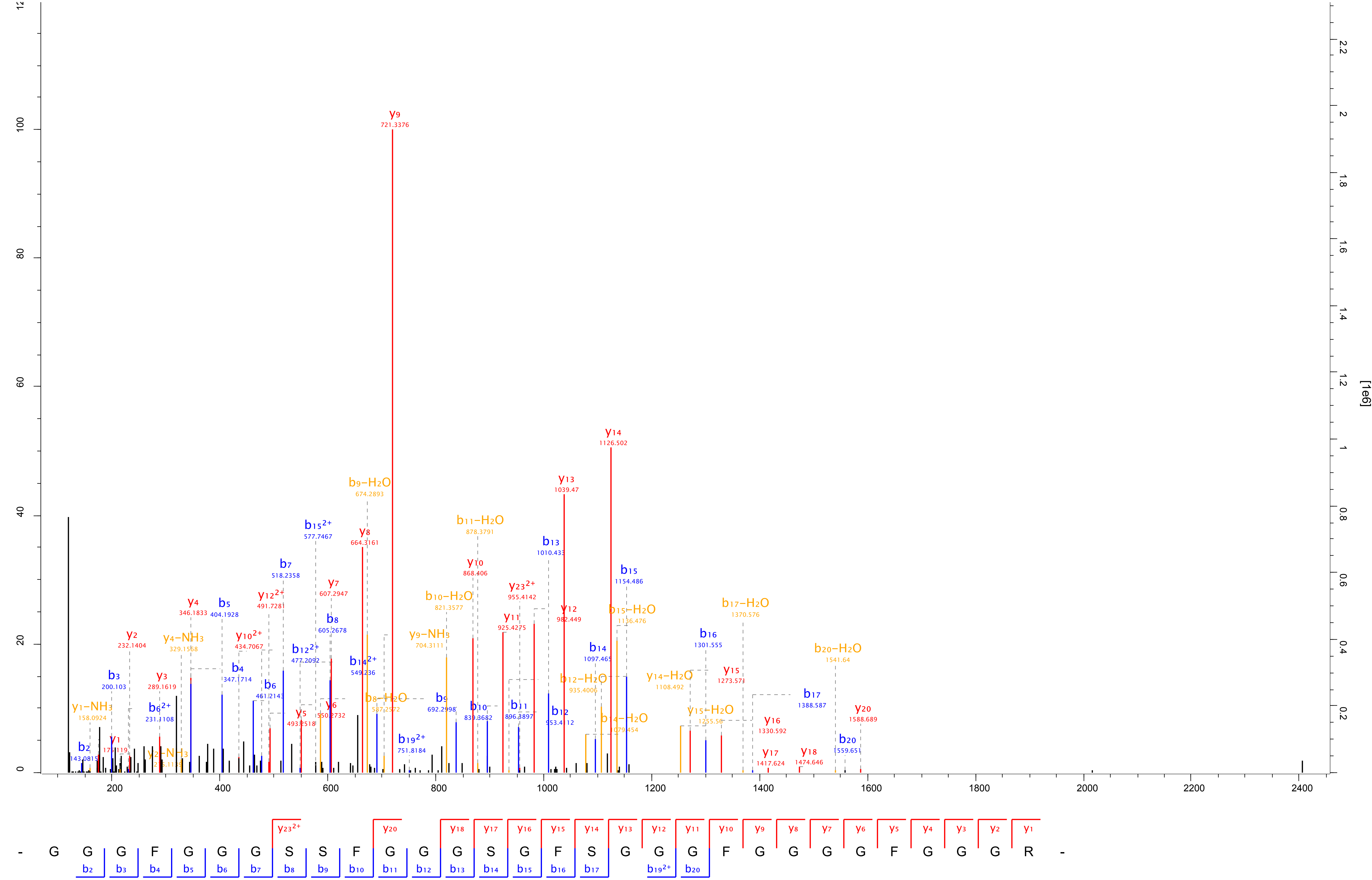

Raw file	Scan	Method	Score	m/z	Gene names
ECS_C1_150508121925	5952	FTMS; HCD	53.45	455.25	YWHAZ

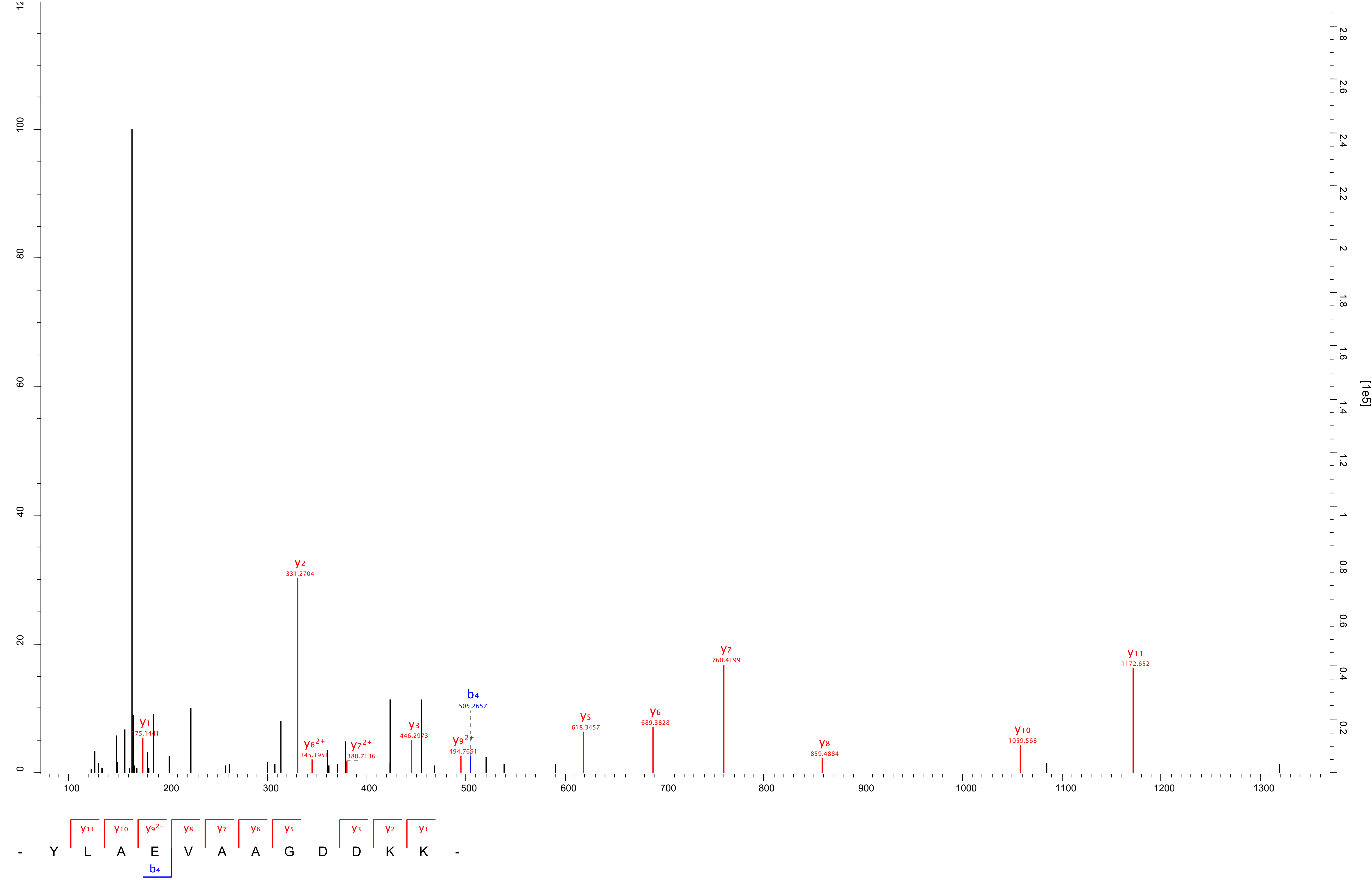

Raw file	Scan	Method	Score	m/z	Gene names
ECS_1B	10309	FTMS; HCD	37.61	450.57	RPS27

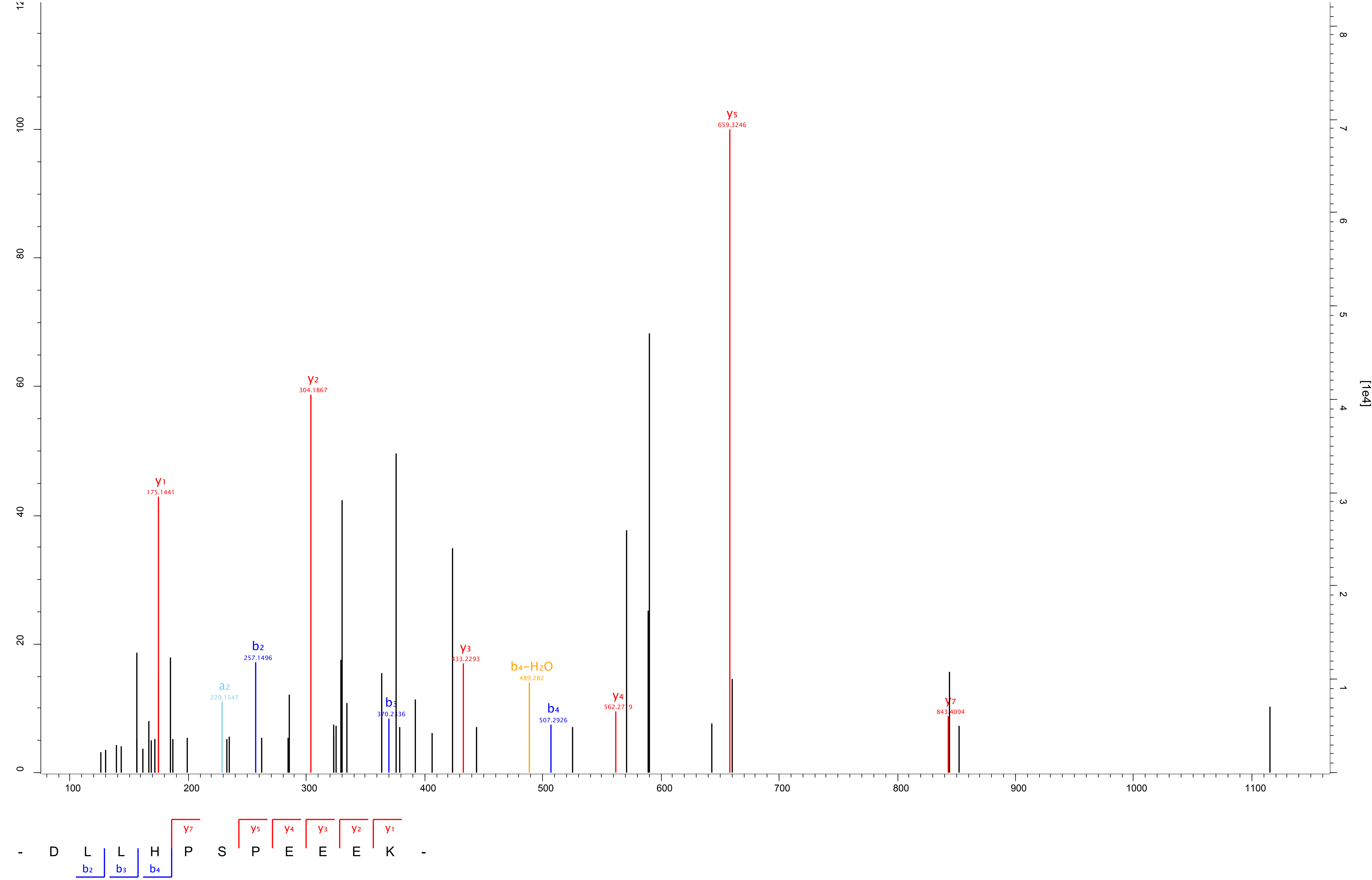

Raw file	Scan	Method	Score	m/z	Gene names
ECS_A1_150507150614	3976	FTMS; HCD	45.83	375.88	NME1-NME2;NME2;NME2P1

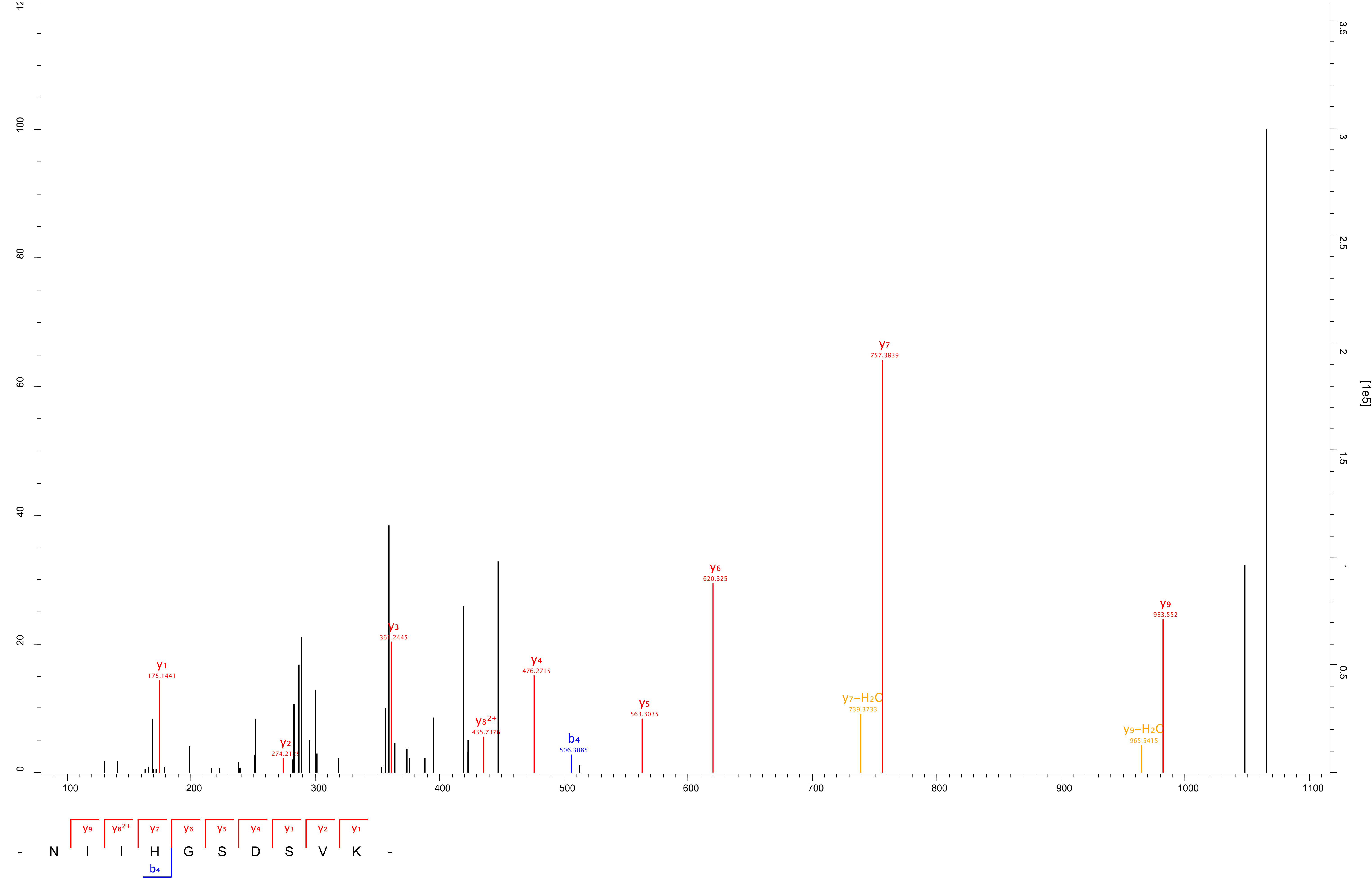

Raw file	Scan	Method	Score	m/z	Gene names
ECS_A3_150507195619	3387	FTMS; HCD	47.71	453.57	KRT31

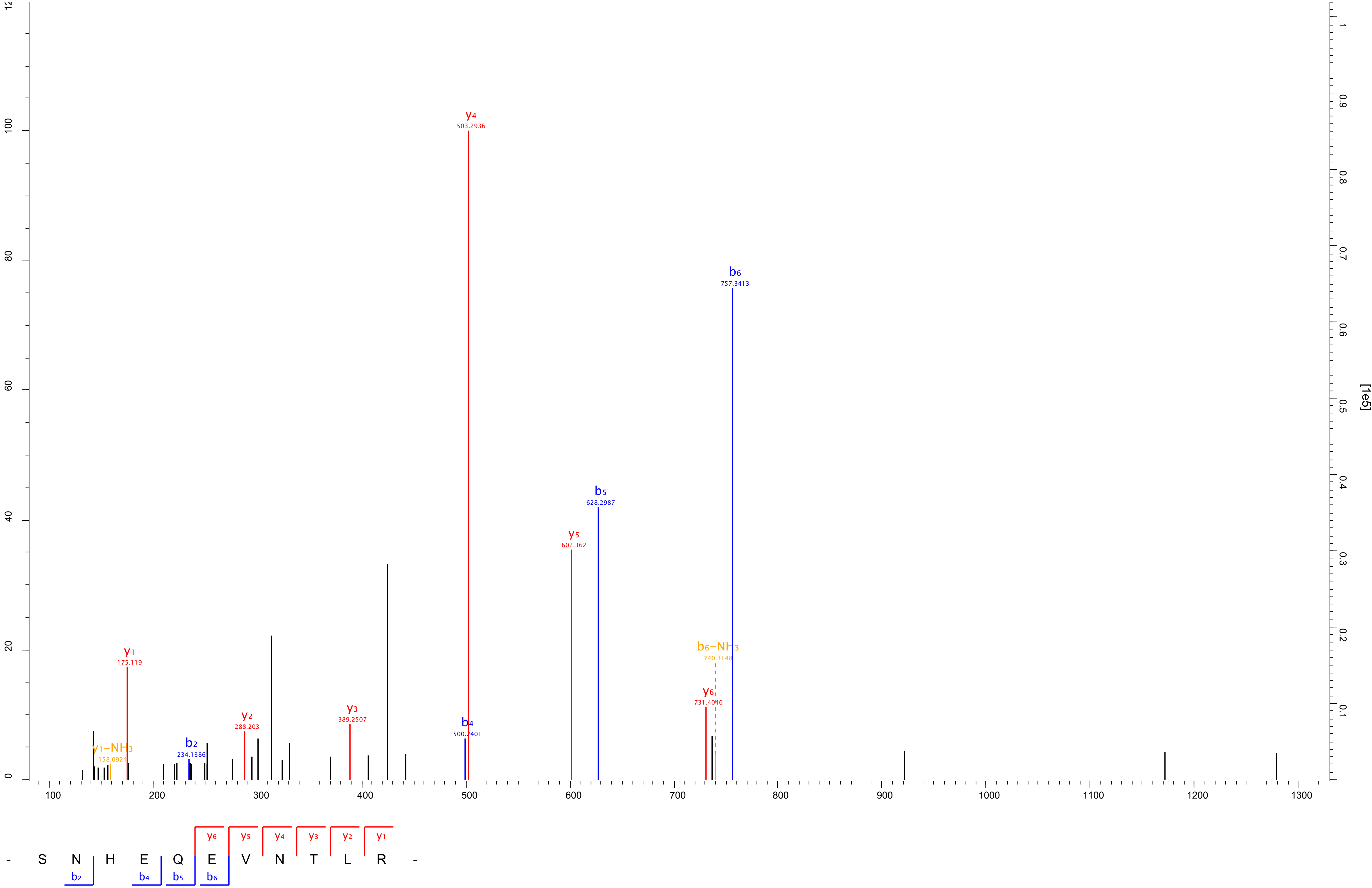

Raw file	Scan	Method	Score	m/z	Gene names
ECS_A1_18_05	9551	FTMS; HCD	81.62	628.83	HSPA7;HSPA6

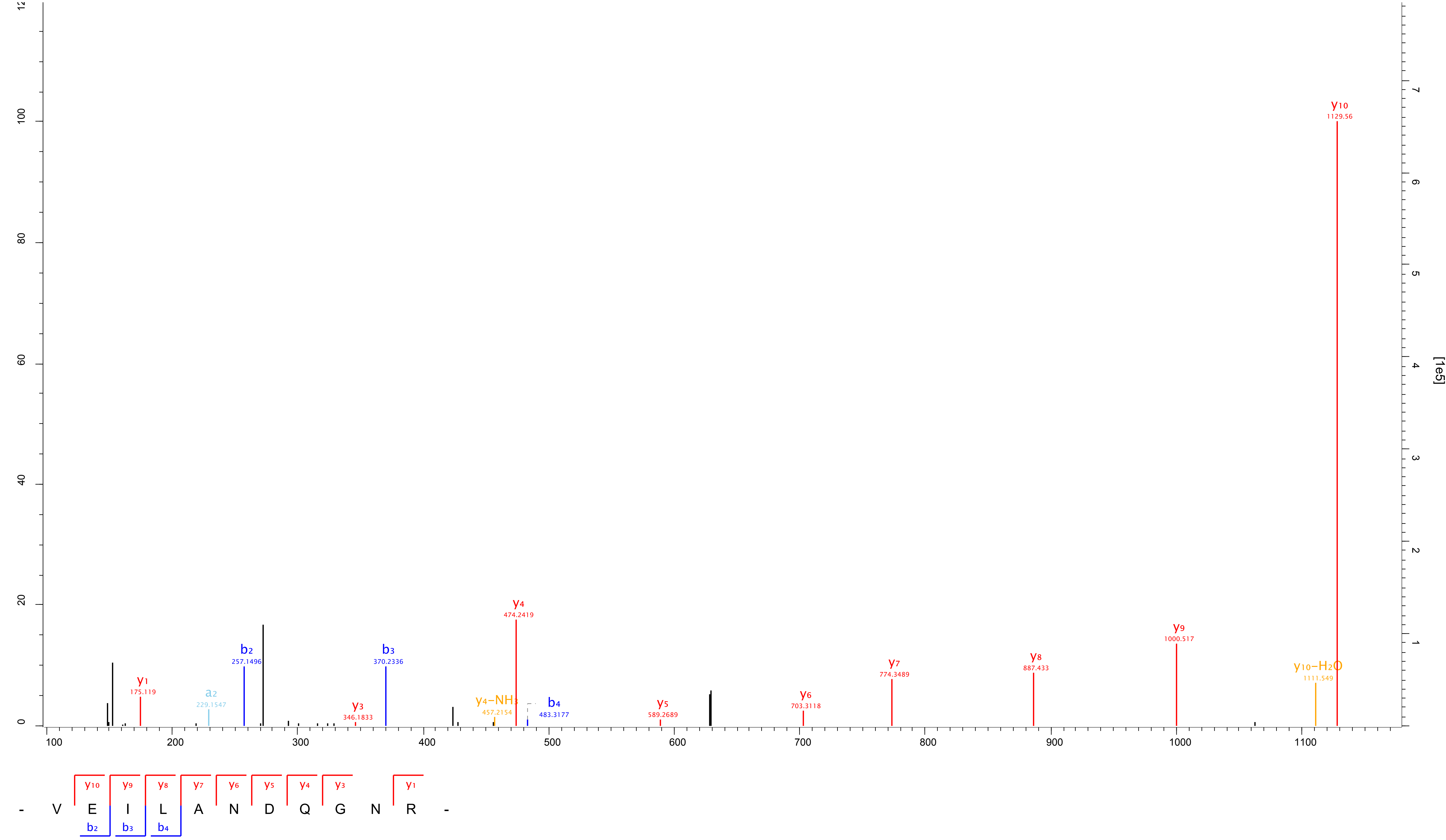

Raw file	Scan	Method	Score	m/z	Gene names
ECS_A2_18_05	20419	FTMS; HCD	117.53	810.36	KRT2

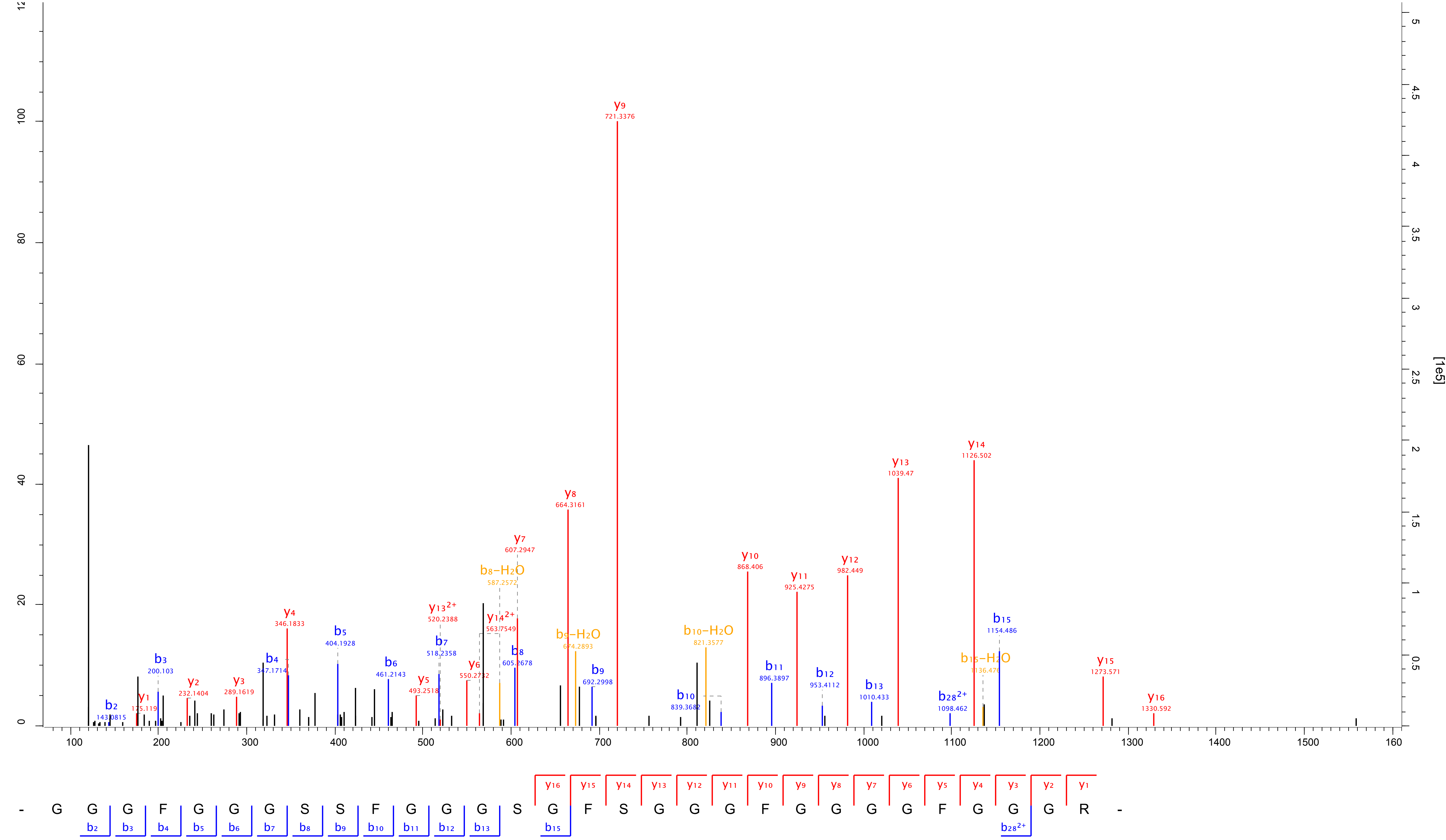

Raw file	Scan	Method	Score	m/z	Gene names
ECS_A2_18_05	12584	FTMS; HCD	75.91	752.42	KRT6C;KRT6A

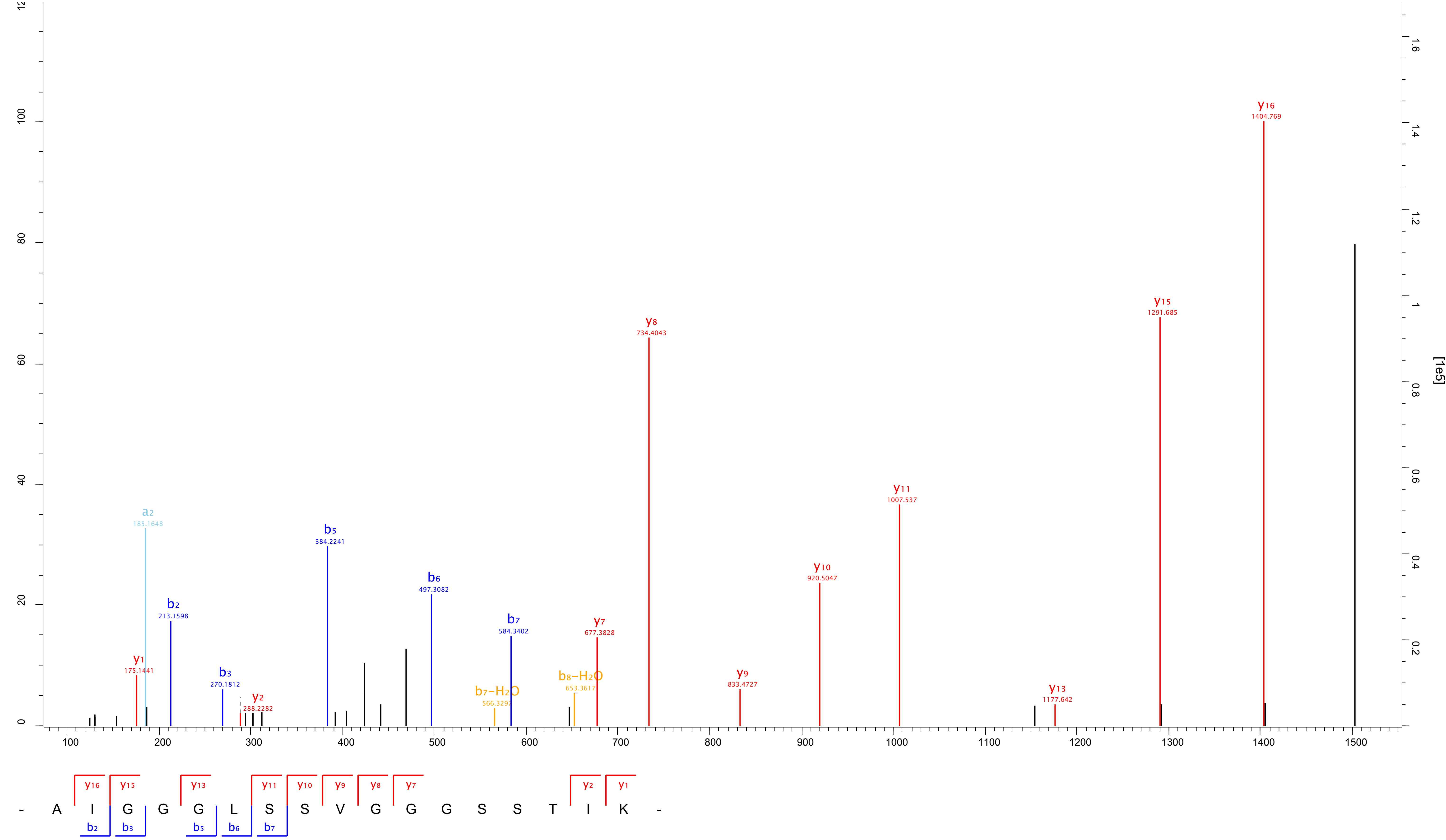

Supplement: Supplemental Data [file 10.1074_M115.700930_jbc.M115.700930-1.pdf]
