# Supplementary material for: The Rab-binding Profiles of Bacterial Virulence Factors during Infection
Source: J Biol Chem. 2016 Jan 11;291(11):5832–43. doi: 10.1074/jbc.M115.700930 (PMC4786718; doi:10.1074/jbc.M115.700930)
Supplement: Supplemental Data [file 10.1074_M115.700930_jbc.M115.700930-2.docx]

**Supplementary Information**

**Supplementary Table 1. Bacterial strains used in this study.**

| **Strain** | **Serogroup/ genotype** | **Reference** |
| --- | --- | --- |
| ***L. pneumophila*** |  |  |
| 130b  (ATCC BAA-74) | O1; clinical isolate | ([1](#_ENREF_1),[2](#_ENREF_2)) |
| ***E. coli*** |  |  |
| TOP10 | *F- mcrA* Δ(mrr-hsdRMS-mcrBC) *φ80lacZ*Δ*M15 nupG recA1 araD139* Δ(ara-leu)*7697 rpsL*(Str^R^) *endA1 λ^-^* | Invitrogen |

**Supplementary Table 2. Plasmids, cloning primers and restriction sites used in this study.**

| **Plasmid** | **Description** | | **RS** | **Source or Reference** |
| --- | --- | --- | --- | --- |
| **pICC562** | **pMMB207c-HA_4_; Vector for the expression of proteins with four N-terminal HA-tags in *L. pneumophila*** | |  | ([3](#_ENREF_3)) |
| pICC562  *-derived* | *Expressed protein* | *Primers 5’-3’* |  |  |
| pICC1565 | HA_4_-SidM |  |  | ([4](#_ENREF_4)) |
| pICC1935 | HA_4_-SidC_PI4P_ | gtcataGGATCCaaatattcctccaagccattattgg | BamHI | This study |
|  |  | ggctaTCTAGActatttctttataactcccgtgtac | XbaI |  |
| pICC1936 | HA_4_-LidA | gcactgaGGTACCGCAAAAGATAACAAATCACATCAAG | KpnI | This study |
|  |  | gtcgcaTCTAGATTATGATGTCTTGAATGGAGATAAAG | XbaI |  |
|  |  | |  |  |
|  |  | |  |  |
| **pICC1544** | **pMMB207c-His_6_-Bio; Vector for the expression in *L. pneumophila* of proteins with 2 N-terminal hexahistidine tags and a BirA biotinylation sequence** | |  | ([4](#_ENREF_4)) |
| *pICC1544-derived* | *Expressed protein* | *Primers 5’-3’* |  |  |
| pICC1937 | His_6_-Bio-SidM | TCGCACTGAGGTACCATGAGTGTTAATGAAGAGCAATTTG | KpnI | This study |
|  |  | GACGTCGCATCTAGATTATTTTATCTTAATGGTTTGTCTTTCTTG | XbaI |  |
| pICC1938 | His_6_-Bio-LidA | as for pICC1936 |  | This study |
|  |  |  |  |  |
| **pICC1939** | **pMMB207c-His_6_-Bio K/A; pMMB207c-His_6_-Bio derivative in which the lysine to which the biotin is attached is mutated to alanine.**  Cloned from pMMB207c-His_6_-Bio (pICC1544) by site directed mutagenesis  CATCTTCGAGGCCCAGGCGATCGAGTGGCACGAG;  CTCGTGCCACTCGATCGCCTGGGCCTCGAAGATG | |  | This study |
| *pICC1939-derived* | *Expressed protein* | *Cloning method* |  |  |
| pICC1940 | His_6_-Bio K/A-SidM | As for pICC1937 |  | This study |
| pICC1941 | His_6_-Bio K/A-LidA | As for pICC1936 |  | This study |
|  |  |  |  |  |
| **pMXs-IRES-Puro** | **pMXs-IP; Viral transduction vector for the expression of proteins in mammalian cells** | |  | Clontech |
| *pMXs-IP-derived* | *Expressed protein* | *Primers 5’-3’* |  |  |
| pICC1942 | GFP-Rab2a | TACTGGATCCGCCACCATGGTGAGCAAGGGCGAGGAG | BamHI | This study |
|  |  | TACTTACTTAGCGGCCGCTCAACAGCAGCCTCCCCC | NotI |  |
| pICC1943 | GFP-Rab5c | TACTGGATCCGCCACCATGGTGAGCAAGGGCGAGGAG | BamHI | This study |
|  |  | TACTTACTTAGCGGCCGCTCAGTTGCTGCAGCACTGGCT | NotI |  |
| pICC1944 | GFP-Rab10 | TACTGGATCCGCCACCATGGTGAGCAAGGGCGAGGAG | BamHI | This study |
|  |  | TACTTACTTAGCGGCCGCTCAGCAGCACTTGCTCTTCCAGCC | NotI |  |
| pICC1945 | GFP-BirA | CTAGGGATCCGCCACCATGGTGAGCAAGG | BamHI | This study |
|  |  | TCTAGCGGCCGCTTATTTTTCTGCACTACGCAGGGA | NotI |  |

**Supplementary Table 3. Crosslinking solutions.**

| **Crosslinking solution** |  |
| --- | --- |
| 1/3% formaldehyde | 1/3% formaldehyde (from 16% solution) (Agar Scientific)  Dulbecco’s PBS (Sigma) |
| DSP | 1mM DSP (from 40mM stock in DMSO) (Pierce)  Dulbecco’s PBS |
| DTME | 0.5mM DTME (from 20mM stock in DMSO) (Pierce)  Dulbecco’s PBS |
| SMCC | 1mM SMCC (from 20mM stock in DMSO) (Pierce)  Dulbecco’s PBS |
| DSP+DTME | 1mM DSP (from 40mM stock in DMSO)  0.5mM DTME (from 20mM stock in DMSO)  Dulbecco’s PBS |

**Supplementary Table 4. Affinity purification buffer compositions.**

| **Buffers** |  |  |  |
| --- | --- | --- | --- |
| Lysis buffer | GnCl/Triton X-100 | RT | 6M guanidium chloride  1% Triton X-100  50mM Na_2_HPO_4_  150mM NaCl  pH 7.3 |
|  | Triton X-100 | 4°C | 1% Triton X-100  50mM Na_2_HPO_4_  150mM NaCl  pH 7.3 |
|  | CHAPS | 4°C | 1% (w/v) 3-[(3-cholamidopropyl)dimethylammonio]-1-propanesulfonate  50mM Na_2_HPO_4_  150mM NaCl  pH 7.3 |
|  | SDS | RT | 0.5% (w/v) sodium dodecyl sulphate  Dulbecco’s PBS |
| His wash buffer |  |  | 1% Triton X-100  50mM Na_2_HPO_4_  150mM NaCl  20mM imidazole  pH 7.3 |
| Elution buffer |  |  | 1% Triton X-100  50mM Na_2_HPO_4_  150mM NaCl  250mM imidazole  pH 7.3 |
| AMBIC |  |  | 50mM ammonium bicarbonate |

**Supplementary Table 5. Dimethyl labelling solution compositions.**

| **Dimethyl labelling solutions** |  |
| --- | --- |
| PB 7.5 | 2ml 50mM Na_2_HPO_4_  7ml 50mM NaH_2_PO_4_ |
| Light labelling solution | 90ul PB7.5  5ul 4% CH_2_O in water  5ul 0.6M NaBH_3_CN in water |
| Heavy labelling solution | 90ul PB7.5  5ul 4% CD_2_O in water  5ul 0.6M NaBH_3_CN in water |

**Supplementary Table 6. Comparison of crosslinker reactivity and linker length.**

| **Crosslinker** | **Reactivity** | **Linker length** |
| --- | --- | --- |
| Formaldehyde | Amine-amine  Lysine-Lysine | 2-3Å |
| DSP | Amine-amine  Lysine-Lysine | 12Å |
| DTME | Thiol-thiol  Cysteine-cysteine | 13.3Å |
| SMCC | Amine-thiol  Lysine-cysteine | 8.3Å |

**Supplementary Figure 1.**

**Supplementary Figure 1.** THP-1 BirA cells were infected with *Legionella* expressing His_6_-Bio-SidM or His_6_-Bio K/A-SidM. A single Neutravidin purification (SAP) was compared with a tandem affinity (Ni^2+^ NTA and Neutravidin) purification (TAP). (A) A heat map showing intensities of all identified proteins found across both SAP and TAP conditions. Proteins were ranked according to SAP enrichment factors. Proteins classified as interactors in either SAP or TAP are in **bold**. (B) A plot of average protein enrichment factors of SAP against TAP conditions. SidM and Rab1A are highlighted by blue and green circles respectively.

**References**

1. Edelstein, P. H. (1986) Control of Legionella in hospitals. *The Journal of hospital infection* **8**, 109-115

2. Engleberg, N. C., Drutz, D. J., and Eisenstein, B. I. (1984) Cloning and expression of Legionella pneumophila antigens in Escherichia coli. *Infection and immunity* **44**, 222-227

3. Dolezal, P., Aili, M., Tong, J., Jiang, J. H., Marobbio, C. M., Lee, S. F., Schuelein, R., Belluzzo, S., Binova, E., Mousnier, A., Frankel, G., Giannuzzi, G., Palmieri, F., Gabriel, K., Naderer, T., Hartland, E. L., and Lithgow, T. (2012) Legionella pneumophila secretes a mitochondrial carrier protein during infection. *PLoS pathogens* **8**, e1002459

4. Mousnier, A., Schroeder, G. N., Stoneham, C. A., So, E. C., Garnett, J. A., Yu, L., Matthews, S. J., Choudhary, J. S., Hartland, E. L., and Frankel, G. (2014) A new method to determine in vivo interactomes reveals binding of the Legionella pneumophila effector PieE to multiple rab GTPases. *mBio* **5**
